# Hydrogels Under Superchaotropic Control: Polyoxometalate Stabilization and pH‐Responsive Crosslinking in Cellulose Ether Solutions

**DOI:** 10.1002/anie.6958664

**Published:** 2026-04-16

**Authors:** Vighnesh B. Lokare, Amina Ledinic, Nina Wehr, Max Hohenschutz

**Affiliations:** ^1^ Institute of Physical Chemistry RWTH Aachen University Landoltweg Aachen Germany; ^2^ Institute of Technical and Macromolecular Chemistry RWTH Aachen University Worringerweg Aachen Germany

**Keywords:** hydrogel, pH stability, polyoxometalate, sol‐gel, superchaotropic

## Abstract

Polymer‐polyoxometalate (POM) systems represent an emergent class of functional hybrid materials. However, the pH‐dependent stability of POMs limits their scope in water. We show here that the water‐mediated, so‐called superchaotropic binding of α‐Keggin POMs to a non‐ionic biopolymer, hydroxypropylcellulose (HPC), (i) selectively stabilizes superchaotropic POMs in water, and (ii) enables pH‐responsive HPC solutions and hydrogels. Raman and NMR spectroscopy revealed that binding to HPC protects the superchaotropic [PW_12_O_40_]^3−^ and [SiW_12_O_40_]^4−^ against hydrolysis, extending their stability from acidic to near‐neutral pH. In turn, POMs with higher charge, stronger hydration, and thus without the ability to bind to HPC, such as [H_2_W_12_O_40_]^6−^, [PW_11_O_39_]^7−^ and [SiW_11_O_39_]^8−^, do not get stabilized. Cloud points, small‐angle neutron scattering, and rotational rheology showed that pH‐induced conversion from superchaotropic [PW_12_O_40_]^3−^ to non‐superchaotropic [PW_11_O_39_]^7−^ switches HPC from a bound, crosslinked to an unbound, non‐crosslinked state, enabling pH‐switchable viscosity and gel‐sol transitions. Superchaotropic stabilization and pH‐switching are proposed as general phenomena in superchaotropic POM/solute systems, highlighting the potential of superchaotropicity in aqueous soft materials.

## Graphical abstract

Superchaotropic binding of Keggin polyoxometalates (POMs) to non‐ionic biopolymers like cellulose ethers (CEs) stabilizes otherwise hydrolytically unstable POMs in aqueous solution and allows conversion of pH‐nonresponsive polymers into pH‐responsive soft materials. The pH‐driven conversion from plenary to lacunary POMs switches superchaotropic binding “on & off,” enabling reversible gel‐to‐sol transitions and tunable viscosity via a novel supramolecular mechanism.

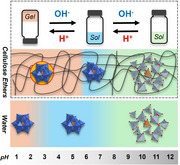

## 1 Introduction

Hybrid materials combining functional inorganic building blocks with organic soft matter offer unique functionalities unattainable in purely organic or inorganic systems [1, 2]. An important class of functional inorganics are polyoxometalates (POMs), anionic oxo‐metal clusters composed of early transition metals (V, W, and Mo) in their highest oxidation states [3, 4]. Their unique structural, electronic, and acidic properties have enabled applications in catalysis [5], energy [6], medicine [7], environmental science [8], and materials science [2]. Over the last two decades, these properties have been harnessed in hybrid materials by immobilizing POMs into soft matter matrices, particularly polymers, resulting in functional self‐assemblies such as hydrogels [9], vesicles [10], sheets [11], membranes [12], and micelles [13]. Such POM/polymer hybrid materials exhibit synergistic behavior, for example, by combining the catalytic properties of POMs with the deformable and responsive structure of polymers [14, 15, 16].

However, the scope of POM/polymer hybrids remains limited due to two fundamental challenges: (i) the immobilization of POMs on polymers is often inefficient and (ii) the stability of POMs in aqueous systems is constrained to highly acidic pH [1, 17]. Immobilization via electrostatic linkage is facile but requires cationic polymers, is often prone to POM leaching, and reduces the aqueous solubility of the resulting material [18, 19, 20]. In contrast, covalent linkage relies on time‐consuming syntheses and compromises the physicochemical properties of POMs [21, 22, 23]. In particular, the hydrolytic instability of POMs restricts the application of hybrids in biological and aqueous systems [24, 25]. While POM degradation can be mitigated by synthetic modifications [26, 27], organic solvents [28, 29], and solution composition [30, 31, 32], the macromolecular environment may impact POM degradation as well [33]. A systematic understanding of plenary POM stabilization and their speciation in aqueous macromolecular environments is lacking. Developing POM/polymer hybrids that combine facile immobilization with effective stabilization of plenary POM in aqueous media promises to extend the applicability of functional POM‐based soft materials.

In the last decade, nanometer‐sized anions (nano‐ions), such as Keggin‐type POMs, have been shown to spontaneously associate with weakly hydrated non‐ionic soft matter systems such as polymers [34, 35], microgels [36, 37], surfactants [38, 39], membranes [40, 41], proteins [42, 43], and hydrotropes [32, 44]. This binding is driven by a water‐mediated phenomenon known as the “superchaotropic effect” [45, 46]. The effect originates from the favorable release of hydration water from the weakly hydrated POMs and the non‐ionic solute, enabling an enthalpic recovery of water‐water hydrogen bonds in the bulk phase [47]. The weak hydration of the POMs arises from their large size and low volume charge density, favoring their association with non‐ionic interfaces [39, 47, 48]. Consequently, this behavior has extended the classical Hofmeister series of ions to various superchaotropic POM types like Keggin, Dawson, Anderson‐Evans, Keplerate, and ring‐shaped species [39, 49, 50, 51]. POM binding to soft matter exceeds that of classical chaotropic ions, such as SCN^−^ and I^−^, and can even exceed that of hydrophobic solutes [38, 45]. Superchaotropic binding typically becomes weaker upon heating, while hydrophobic binding, which is enthalpically unfavorable, becomes stronger upon heating due to opposite thermochemical binding signatures [52]. Therefore, superchaotropic POM binding offers an effective supramolecular immobilization strategy for creating soft hybrid materials.

Modern soft materials like hydrogels have shifted from static to dynamic “smart” materials [53]. Smart hydrogel systems can respond reversibly to different stimuli such as light, pH, electric fields, and temperature, thereby eliciting dramatic changes in their properties such as viscosity [54], sol‐gel transitions [55], color [56], conductivity [57], and shape [58]. Supramolecular interactions [59] like electrostatics, hydrophobic, π–π, host‐guest complexation, and hydrogen bonding have been widely exploited to yield hydrogels with adaptive behavior, leading to applications in biomedicine [60], soft robotics [61], and sensing [62]. While responsive supramolecular hydrogels are mostly based on synthetic polymers or molecular components, growing environmental challenges incentivize sustainable alternatives [63, 64]. Bio‐based polymers offer advantages owing to their low costs, abundant availability, low toxicity, and biodegradability/compatibility [65]. Among them, cellulose ethers (CEs) derived from wood cellulose are a versatile class of “green” macromolecules utilized in diverse applications [66]. We recently demonstrated that superchaotropic binding of Keggin POMs to non‐ionic CEs induces crosslinking, enabling the facile formation of hydrogels [34]. Earlier studies had shown that these POMs induce structural changes in shorter synthetic polymers such as pNiPAM and PEG, albeit without gelation [35, 67]. The use of superchaotropic binding as a supramolecular strategy to develop stimuli‐responsive behavior in bio‐polymeric hydrogels has remained unexplored.

**FIGURE 1:**
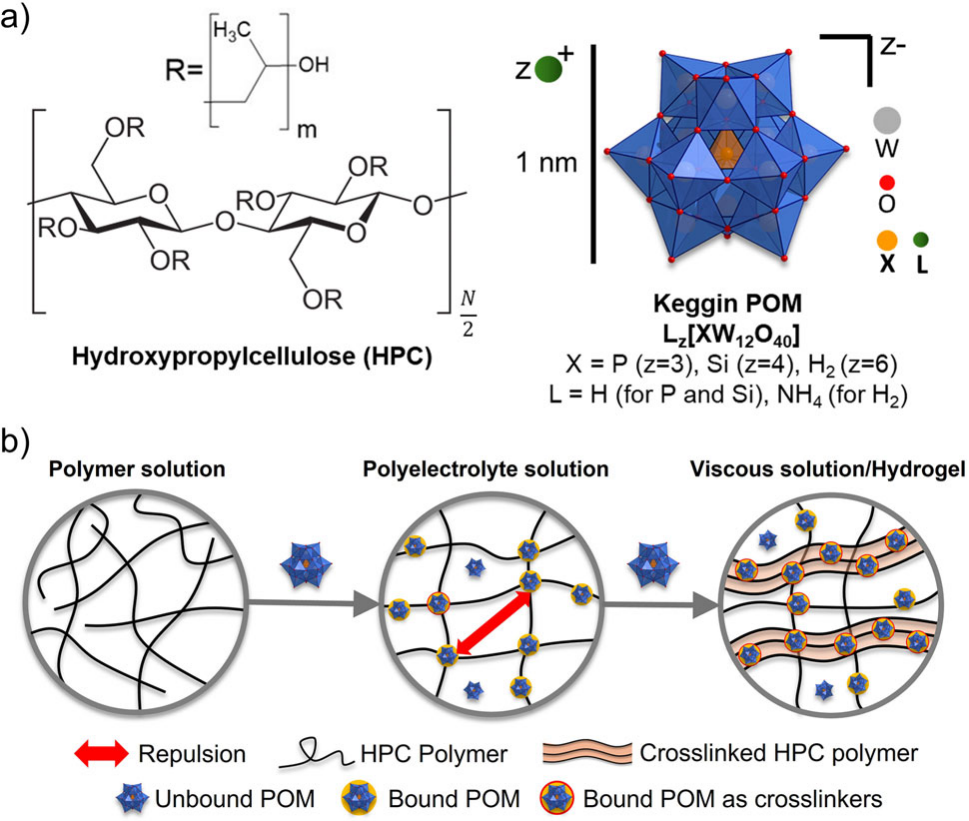
(a) Chemical structures of HPC and Keggin POMs H_3_[PW_12_O_40_] (PW_12_), H_4_[SiW_12_O_40_] (SiW_12_) and (NH_4_)_6_[H_2_W_12_O_40_] (H_2_W_12_). The used HPC has an average of 3.8 hydroxypropyl substituents per glucose unit and a degree of polymerization of *N* = 230. (b) Illustration of the thickening mechanism for POM‐induced crosslinking of HPC due to superchaotropic binding.

In this contribution, we demonstrate for the first time how superchaotropic binding can serve simultaneously (i) as an immobilization strategy that also stabilizes Keggin POMs against hydrolytic decomposition in macromolecular environments and (ii) as a supramolecular design principle for making pH‐responsive hydrogels. For these purposes, we investigated the pH‐dependent speciation of three different Keggin POMs—H_3_[PW_12_O_40_] (PW_12_), H_4_[SiW_12_O_40_] (SiW_12_), (NH_4_)_6_[H_2_W_12_O_40_] (H_2_W_12_)—in the presence of the non‐ionic polymer hydroxypropylcellulose (HPC) (chemical structures shown in Figure 1a). We used two POMs, PW_12_ and SiW_12_, which were previously shown to act as crosslinkers at millimolar concentrations when added to HPC solution, forming viscous solutions or hydrogels [34] (Figure 1b), and compared their speciation behavior to a non‐crosslinking POM H_2_W_12_. We found that superchaotropic POMs such as PW_12_ and SiW_12_ interact with HPC, enhancing POM stability in solution against decomposition, and that their pH‐dependent speciation imparts a reversible pH‐switchable response in HPC solutions, which we further extended to methylcellulose (MC)‐based hydrogels, enabling a gel‐sol‐gel transition. Our work paves the way for the development of POM/soft matter hybrid materials based on superchaotropic binding that combine enhanced POM stability in solution with built‐in pH‐responsive functionality.

## 2 Results and Discussion

### 2.1 Superchaotropic Binding Affects POM Speciation in Aqueous Solution

**FIGURE 2:**
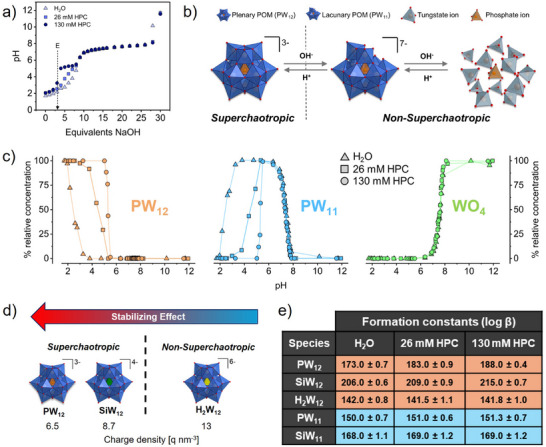
Effect of superchaotropic binding on the pH speciation of Keggin‐type POMs. (a) pH titration of 5 mM PW_12_ in H_2_O and in the presence of 26 and 130 mM HPC. NaOH was added in molar equivalents relative to the PW_12_ concentration. (b) Illustration showing pH‐dependent speciation of plenary Keggin POM ([PW_12_O_40_]^3−^) into lacunary Keggin POM ([PW_11_O_39_]^7−^) and its constituent anions (WO_4_^2−^ and PO_4_^3−^) upon basic hydrolysis and its reversibility by acidic polycondensation. (c) pH‐dependent speciation profiles of 5 mM PW_12_ in H_2_O and in the presence of 26 and 130 mM HPC, obtained using Raman spectroscopy. (d) Schematic showing the dependence of pH stabilization in Keggin POMs by HPC on their charge density and superchaotropic character. (e) Table showing formation constants (log β) of species formed during the decomposition of Keggin POMs in H_2_O and in presence of 26 and 130 mM HPC, calculated by modeling pH speciation profiles obtained from Raman spectroscopy.

Firstly, we used pH titrations to examine the effect of HPC on the decomposition behavior of Keggin POMs in aqueous solution. Figure 2a shows pH titrations of 5 mM PW_12_ as a function of added molar equivalents of NaOH, both in water and in the presence of HPC at 26 and 130 mM. The equilibration of POMs speciation is a slow process [17], taking up to 7 days to reach a stable pH as shown by time‐resolved pH measurements in Figures S1–S3. Hence, here we only present data from samples equilibrated for 2 weeks. POMs behave as strong acids (pKa < 0) that release all their acidic protons in aqueous solution [68, 69]. In pure water, the titration curve of PW_12_ lacks an equivalence point corresponding to complete neutralization of its three acidic protons. Instead, the pH gradually increases up to pH 7. This observation indicates the onset of decomposition of the plenary PW_12_ before all acidic protons are neutralized. The broad buffering region at 7 < pH < 8 indicates further hydrolysis of PW_12_. An endpoint occurs at pH 12, indicating complete decomposition of PW_12_.

Remarkably, the titration profile of PW_12_ changes significantly upon increasing the HPC concentration to 130 mM. An equivalence point (E) appears at three molar equivalents of NaOH that leads into a buffering region at pH ≈ 5, a feature absent in the pure aqueous system and in the presence of 26 mM HPC. The occurrence of this equivalence point signifies neutralization of all three acidic protons of PW_12_ without decomposition of the plenary PW_12_ up to pH 5, implying an improved hydrolytic stability in the presence of HPC. The subsequent buffer region from pH 5 to 5.5 indicates the onset of base‐induced hydrolysis of PW_12_. Above pH 5.5, the pH titration curves for PW_12_ in H_2_O and with HPC overlap, indicating that the stabilizing effect of HPC is restricted to the low pH species of PW_12_. A similar, albeit weaker, stabilizing effect was observed in the titration of SiW_12_, see Figure S4a. In contrast, the titration curve of H_2_W_12_ remained unchanged upon addition of HPC, see Figure S4b, indicating that HPC has no effect on the decomposition behavior of H_2_W_12_ and that HPC does not undergo any acid‐base equilibria itself.

To identify the POM species involved in the pH titration and quantify the stabilizing effect due to HPC, we measured and analyzed Raman spectra of the same samples. We focused on the spectral region of the W–O stretching vibration at 900–1030 cm^−1^ that probes the terminal W═O bonds in the Keggin structure. The Raman bands were deconvoluted into contributions of individual POM species by a sum of Voigt profiles; see details in Section S3.1. Table 1 displays the relevant band positions characteristic of the individual POM species, in close agreement with those reported for Keggin POMs in water [28, 70, 71, 72, 73]. An overview of all fitted peak positions is given in Section S3.2.

**TABLE 1:** Characteristic Raman shifts for different solution species during pH speciation as obtained from Raman spectra.

Species	Anion	Labels	Raman shift (cm^−1^)
Plenary POM	[PW_12_O_40_]^3−^	PW_12_	1010
	[SiW_12_O_40_]^4−^	SiW_12_	998
	[H_2_W_12_O_40_]^6−^	H_2_W_12_	980
Lacunary POM	[PW_11_O_39_]^7−^	PW_11_	960
	[SiW_11_O_39_]^8−^	SiW_11_	960
Tungstate	WO_4_^2−^	WO_4_	930

The plenary POM [PW_12_O_40_]^3−^ (PW_12_) decomposes into lacunary POM [PW_11_O_39_]^7−^ (PW_11_) and phosphate PO_4_^3−^ (PO_4_) and tungstate WO_4_^2−^ (WO_4_) ions upon basic hydrolysis, as shown in Figure 2b. Figure 2c shows the influence of HPC on the stability range of the species involved in speciation of PW_12_ across a pH range of 1 to 12 as obtained from Raman spectroscopy. In pure water, PW_12_ is only stable at pH < 1.7. Addition of NaOH leads to the formation of PW_11_ at 1.7 < pH < 8 and WO_4_ at pH > 6.5. The presence of HPC significantly changes the speciation behavior by delaying the onset of PW_12_ hydrolysis. For 26 mM HPC, PW_12_ starts decomposing at pH 3 and for 130 mM HPC at pH 5, thus enhancing the stability range of PW_12_ by 3.3 pH units. Once the plenary PW_12_ was absent at pH 5.5, HPC had no effect on the subsequent decomposition, in line with the convergence seen in the pH titration (Figure 2a). A comparable effect was observed with SiW_12_ where the stability range was extended by 1.7 pH units in the presence of 130 mM HPC, see Figure S14a. While HPC delayed the formation of lacunary POMs (PW_11_ and SiW_11_), it had no effect on the formation of tungstate ions at high pH. By contrast, the addition of HPC did not alter the decomposition behavior of H_2_W_12_ as shown in Figure S14b. This result was further confirmed by ^1^H NMR measurements of H_2_W_12_ in D_2_O and in the presence of 26 mM HPC in D_2_O (Section S4), with identical decomposition trends. The hydrolysis of H_2_W_12_ to WO_4_ occurred without the formation of a lacunary POM as observed by both Raman and ^1^H NMR spectroscopy. Accordingly, in the presence of HPC, PW_12_ exhibited a stronger stabilization effect than SiW_12_ and H_2_W_12_. Although the three POMs have comparable size and shape, differences in their charge density significantly influence their binding affinity to non‐ionic interfaces [47]. A lower charge on the POM leads to higher superchaotropicity, which is consistent with the pH stabilization following the order: PW_12_ (*z* = −3) > SiW_12_ (*z* = −4) >> H_2_W_12_ (*z* = −6), see Figure 2d. This trend implies that superchaotropic POMs selectively stabilize themselves by binding with HPC. In contrast, the non‐superchaotropic POM H_2_W_12_ does not bind with HPC due to its high charge density and strong hydration, which thermodynamically disfavors its dehydration required for superchaotropic association with HPC. As a result, no stabilization of H_2_W_12_ was observed in the presence of HPC.

We modeled the pH‐dependent speciation profiles to determine formation constants (log β) for plenary and lacunary POMs (Figures S15–S17). The involved formation reactions, formation constants, and mass balance equations used to model the experimental data are detailed in Section S3.4. Figure 2e summarizes the computed formation constants for different POM species in H_2_O, 26 mM HPC and 130 mM HPC. The presence of HPC significantly shifted the plenary‐lacunary thermodynamic equilibrium toward the formation of the superchaotropic plenary POM, while having a negligible effect on the non‐superchaotropic plenary and lacunary POMs. Notably, the formation constant for PW_12_ in H_2_O is increased by 15 orders of magnitude in the presence of HPC.

In complementary experiments, we also studied the pH speciation of PW_12_ in water and in the presence of 26 mM HPC using ^31^P NMR spectroscopy; see Section S5. The effect of HPC on the stable pH range of PW_12_ observed by ^31^P NMR closely agrees with that observed by Raman spectroscopy. Besides the main species PW_12_, PW_11_, and PO_4_, ^31^P NMR additionally identified two intermediate species (PW_11_)WO_2_ and (PW_11_)_2_WO_2_ at pH 3 in D_2_O. The presence of 26 mM HPC significantly stabilized PW_12_, firstly by delaying the onset of PW_12_ hydrolysis but more importantly by fully suppressing the formation of the intermediate species (PW_11_)WO_2_ and (PW_11_)_2_WO_2_.

Notably, Haouas et al. recently reported a similar increase in hydrolytic stability of PW_12_ with the macrocyclic host molecule γ‐cyclodextrin (γ‐CD) [30]. In the presence of two equivalents of γ‐CD, PW_12_ remained the sole species up to pH 4 compared to pH 2 without γ‐CD in D_2_O as shown by ^31^P‐NMR. Herein, we find a stabilization of PW_12_ with 130 mM HPC up to pH 5 in H_2_O, see Figure 2c, and thus an even more efficient stabilization effect. HPC and γ‐CD are chemically similar but differ in the geometry of their binding sites and consequently in their binding affinity [34]. The stabilization of PW_12_ by γ‐CD arises from the formation of 1:1 and 1:2 inclusion complexes, where the size‐matching concave hydrophobic cavity shields PW_12_ against hydrolysis. By contrast, on the linear polymer HPC, the binding site is ill‐defined, and the binding constant is anticipated to be smaller. Regardless, HPC might allow for good protection against hydrolysis due to a wrapping of hydroxypropyl groups around PW_12_ in particular when two polymer chains are involved in the binding site such that PW_12_ acts as a physical crosslink between them. Further, 130 mM HPC represents 26 hydroxypropyl glucose units per PW_12_ compared to 16 glucose units per PW_12_ by Haouas et al. This difference in host excess leads to a higher concentration of unoccupied binding sites with HPC that shifts the binding equilibrium toward bound PW_12_ by Le Chatelier's principle and thus further contributes to the efficient stabilization with HPC.

### 2.2 Probing POM Binding and HPC Solution Structure During Speciation of the POMs

A previous study showed that the binding of superchaotropic POMs SiW_12_ or PW_12_ drastically affects the solution structure of HPC by inducing an interplay of (i) long‐range electrostatic repulsion between polymer chains due to bound POMs and (ii) short‐range chain‐chain attraction due to bound POMs acting as physical crosslinks [34]. Base‐induced decomposition of these POMs is anticipated to dramatically weaken superchaotropic binding because the decomposed POM species show increased electric charge (density) and therefore favorable hydration compared to the plenary superchaotropic POM, as calculated according to a Born solvation‐surface model in Section S6 [48]. Accordingly, as the pH increases and the POM decomposes, the associated polymer structure arising from superchaotropic binding should also progressively disappear.

**FIGURE 3:**
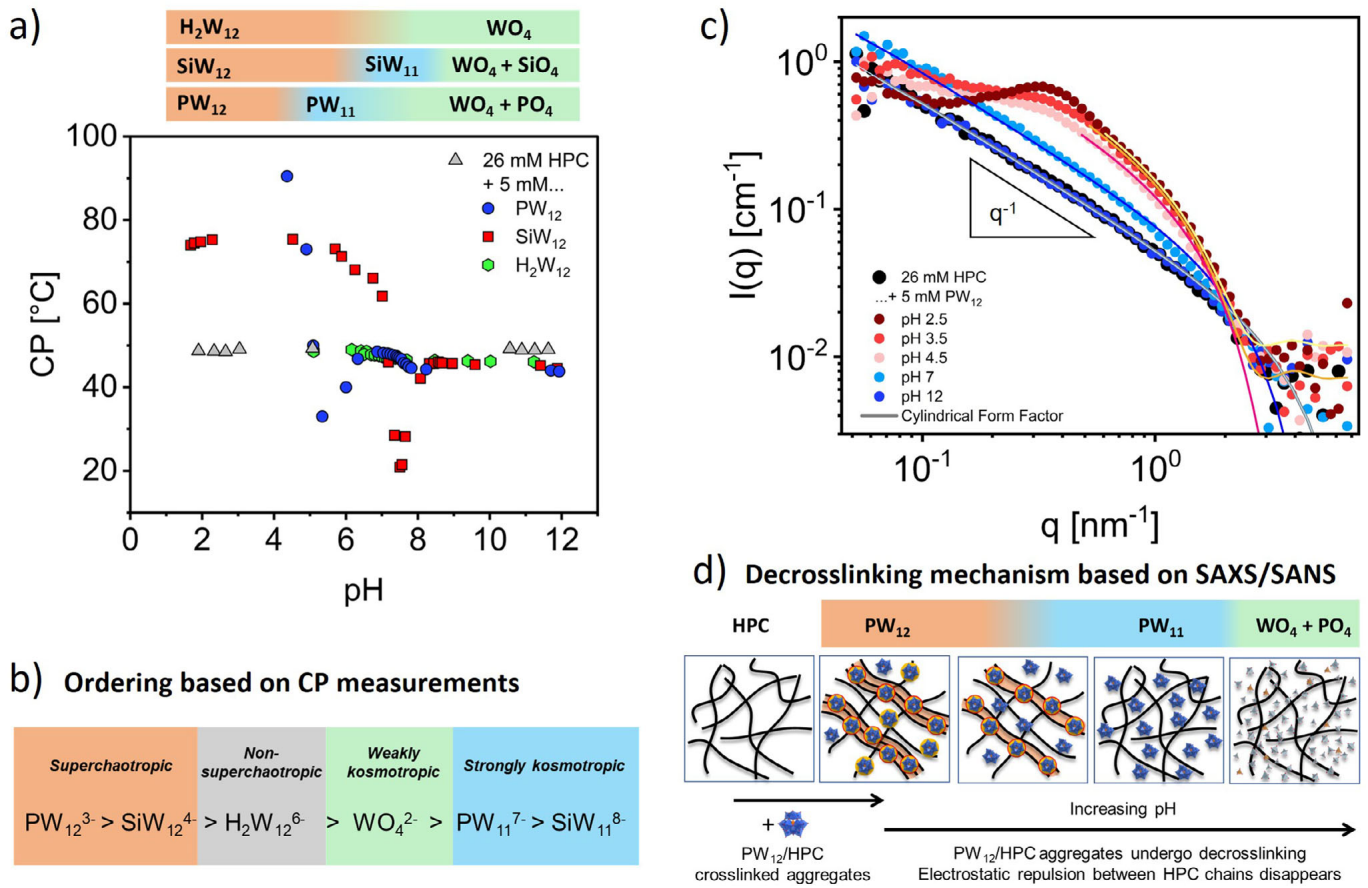
Solution structure of HPC during pH speciation of Keggin‐type POMs. (a) Cloud point (CP) evolution of 26 mM HPC as a function of pH without POM and with 5 mM Keggin POMs (PW_12_, SiW_12_, H_2_W_12_). Top: Speciation diagram showing solution species as a function of pH. Labels: SiO_4_ for SiO_4_^4−^ and PO_4_ for PO_4_^3−^. (b) Ranking of the observed POM species during pH speciation according to their (super)chaotropic/kosmotropic character (based on cloud point measurements). (c) SANS profile at various pH for 26 mM HPC with 5 mM PW_12_ in D_2_O. Solid lines represent fits for the data (see text). (d) Scheme showing mechanism for pH‐driven decrosslinking of PW_12_/HPC aggregates based on the data obtained from SAXS/SANS.

To elucidate the effects of POM speciation on the binding to HPC and the resulting HPC solution structure, we performed cloud point (CP) measurements, small‐angle x‐ray scattering (SAXS), and small‐angle neutron scattering (SANS) as a function of pH. CP measurements serve as a simple tool to assess the impact of solutes on the interactions between polymer chains. For polymers with a lower critical solution temperature, such as HPC, hydration becomes weaker upon heating, which leads to chain collapse and aggregation that appears as visual turbidity at the CP temperature. Added salts can either promote HPC aggregation and decrease the CP (kosmotropic, salting‐out effect) or inhibit HPC aggregation and increase the CP (chaotropic, salting‐in effect) [74]. A CP‐decrease informs on additional attractive forces between the polymer chains, while a CP‐increase informs on additional repulsive, stabilizing forces between the polymer chains. Addition of superchaotropic POMs has been shown to dramatically increase the CP of non‐ionic polymers [52] and surfactants [47] related to binding and electric charging of the non‐ionic solute, which inhibits aggregation of the solute by electrostatic repulsion [39, 75]. The extent of CP increase allowed ranking the superchaotropicity of different Keggin POMs [47]. Figure 3a shows changes in the CP of 26 mM HPC as a function of pH without POM and with 5 mM of each Keggin POM, correlated with their speciation diagram shown at the top of the plot. The CP of 26 mM HPC without added POM is constant at 49°C for 2 < pH < 12. Changes in pH therefore affect the CP exclusively through changes in the POM species. At low pH, PW_12_ and SiW_12_ are present and dramatically increase the CP of HPC. Below pH 4, the CP with PW_12_ is above the boiling point of water and thus outside the measurable range. The CP then drops above pH 4, where the hydrolysis of PW_12_ to lacunary PW_11_ is initiated. The CP is higher with PW_12_ than with SiW_12_, implying stronger superchaotropic binding of PW_12_ to HPC. In contrast, H_2_W_12_ had no effect on the CP, indicating negligible interaction with HPC. The series of plenary POMs according to their CP‐increase: PW_12_ > SiW_12_ > H_2_W_12_ confirms the previously established order according to the POMs superchaotropicity [76]. For PW_12_, the CP decreases sharply from pH 4 to 5, as the plenary PW_12_ decomposes to lacunary PW_11_. The CP reaches a minimum at pH 5.3, coinciding with the maximum abundance of PW_11_ and complete disappearance of PW_12_. This minimum occurs due to the strong kosmotropic nature of the lacunary POM, which, due to its very high charge, *z* = −7, acts as a salting‐out agent that competes for hydration water with HPC. From pH 5.3 to 7, the CP increases as the lacunary POM converts to kosmotropic phosphate and tungstate ions. Upon complete hydrolysis of PW_12_ above pH 8, the CP plateaus at a value of 44°C slightly below the CP of HPC without POM (49°C), as only weakly kosmotropic species remain.

Similar trends in CP are observed for the speciation of SiW_12_, where the conversion of superchaotropic plenary SiW_12_ to strongly kosmotropic lacunary SiW_11_ is accompanied by a minimum at pH 7.5, followed by an increase and plateauing of the CP value on the formation of weakly kosmotropic tungstate and silicate ions. The CP minimum observed in the presence of SiW_11_ is lower than that with PW_11_. This suggests a greater salting‐out capacity and kosmotropicity of the lacunary SiW_11_ compared to PW_11_, owing to its higher charge z = ‐8. Moreover, the CP during the pH speciation of H_2_W_12_ shows no significant change, which reflects the non‐superchaotropic and weakly kosmotropic behavior of the two involved species: H_2_W_12_ and WO_4_, respectively. These CP measurements allowed ranking the involved POM species according to their chaotropic/kosmotropic character, as shown in Figure 3b.

SANS and SAXS measurements were performed to reveal the pH‐dependent structural evolution of HPC and POM/HPC aggregates at the nanometer scale. We used HPC at the overlap concentration (c* = 1% w/w, 26 mM), as this maximizes the scattered intensity while measuring the well‐defined form factor of individual polymer chains and avoiding less‐defined entangled structures at higher polymer concentrations. The SANS profiles of HPC in the presence of 5 mM PW_12_ at different pH values are shown in Figure 3c. For SANS measurements, D_2_O was used as the solvent to maximize contrast with hydrogenated HPC. The scattering contrast of D_2_O matches that of POM species, making them effectively “neutron transparent”. Consequently, only the HPC chains and POM/HPC aggregates produce scattering. In SANS, the scattering of 26 mM HPC increases monotonically with a *q* ^−1^ dependence, as typical for a semi‐flexible polymer. Fitting the data with a cylindrical form factor (details of the fitting procedure are discussed in Section S7.1) yields a radius of 0.46 nm, consistent with the expected radius for a cellulose polymer. Upon addition of PW_12_ at pH 2.5, a pronounced increase in the scattering intensity and appearance of a correlation peak at q* = 0.3 nm^−1^ is observed, corresponding to a long‐ranged HPC‐HPC distance of 20 nm. The increased intensity indicates formation of physically crosslinked PW_12_/HPC aggregates with a cylinder radius of 1.3 nm. Meanwhile, the correlation peak arises from the electrostatic repulsion between HPC chains electrically charged by adsorbed PW_12_. As the pH increases from 2.5 to 4.5, PW_12_ partially hydrolyzes to non‐adsorbing PW_11_, resulting in decreased scattering intensity due to disassembly of PW_12_/HPC aggregates. The correlation peak broadens into a shoulder, indicating weakened interchain repulsion due to a decrease in PW_12_ binding and enhanced electrostatic screening by PW_11_. The correlation peak disappears completely at pH 7, indicating no chain‐chain repulsion, consistent with PW_11_ being the sole species at this pH. However, the scattered intensity at pH 7 is higher than that of HPC without POM, which indicates some remaining chain aggregation. pH titration and Raman spectroscopy here showed that PW_12_ is stable towards higher pH in D_2_O than in H_2_O, see Section S3.5, related to a higher bond dissociation energy of D‐O bonds compared to H‐O bonds [84], which rationalizes the observed chain aggregation. However, the SAXS profile for PW_12_/HPC in H_2_O at pH 5.4 was described as a sum of the individual intensities of HPC and PW_11_, see Figure S27, indicating that both species are structurally uncorrelated. At pH 12, the scattering profile exactly matches that of 26 mM HPC, indicating that HPC is non‐aggregated in solution and that tungstate and phosphate, present at this pH, have no interaction with HPC. Fitting of SANS profiles with a cylindrical form factor revealed a decrease in the aggregate radius from 1.3 nm at low pH to 0.46 nm at high pH, confirming the pH‐dependent decrosslinking of PW_12_/HPC aggregates. Overall, SANS and SAXS measurements consistently show that PW_12_ crosslinks HPC at low pH, whereas a pH‐dependent hydrolysis to PW_11_ and smaller anions at higher pH results in decrosslinking of the PW_12_/HPC aggregates into individual HPC chains, as shown in Figure 3d. Similar trends were observed for SiW_12_ with HPC; see Section S7.5.

The SAXS, SANS, and CP experiments show that exclusively superchaotropic POMs (PW_12_ and SiW_12_) bind to HPC, while their decomposition products do not. This result allows rationalizing the stabilization of POMs by coupled equilibria; see Section S8. Hydrolysis of PW_12_ and SiW_12_ thus exclusively occurs in their unbound state, while POM that is bound to HPC does not hydrolyze. The POM‐HPC binding equilibrium depletes the solution of POM, which shifts the hydrolysis equilibrium toward higher OH^−^ concentration and higher pH, and thus leads to superchaotropic POM stabilization.

### 2.3 Translating Speciation Into Function: pH Switchable Superchaotropic Materials

**FIGURE 4:**
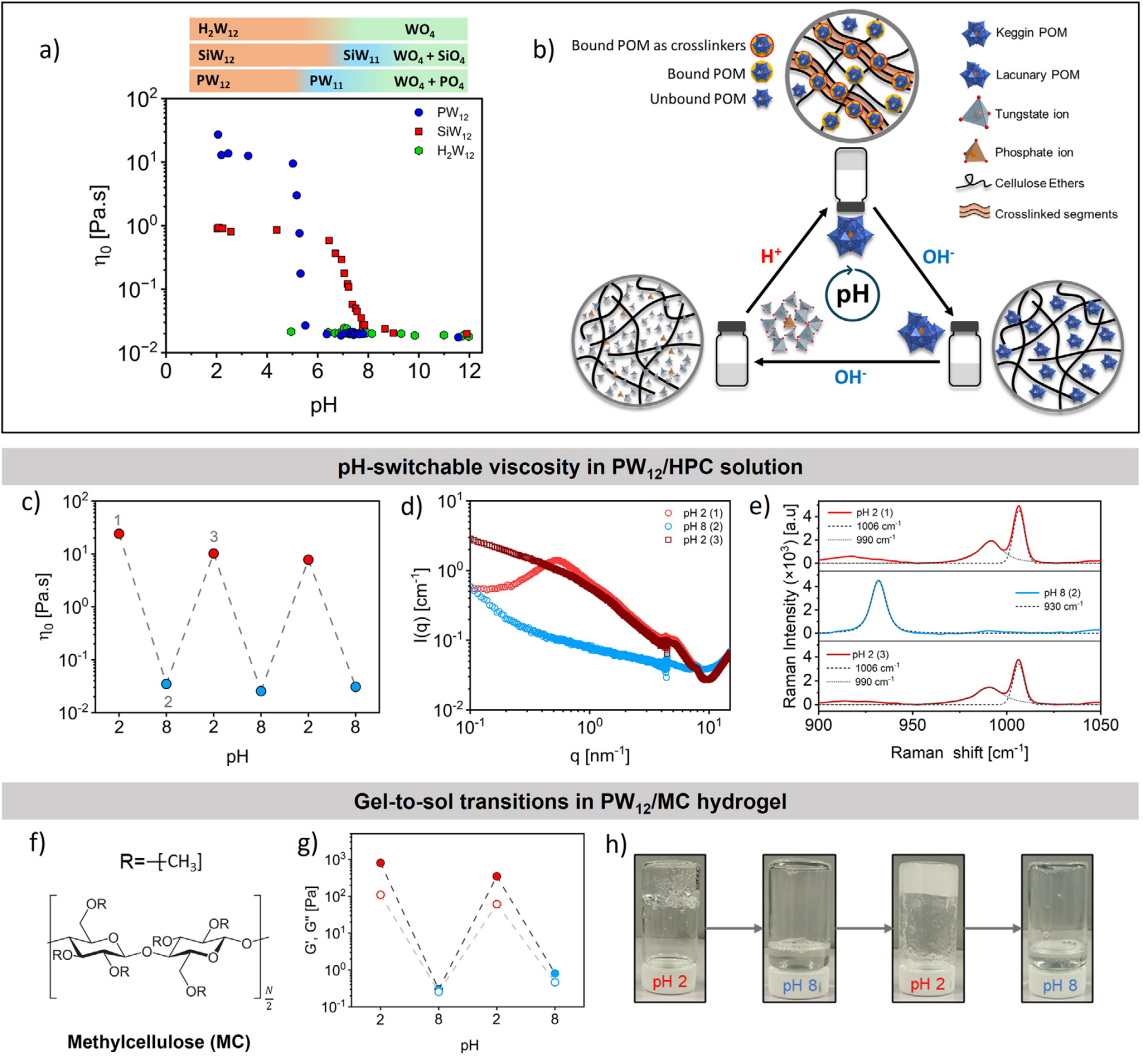
Rheology and pH‐switchable behavior for cellulose ethers/POM solution and hydrogel. (a) Zero‐shear viscosity of 130 mM HPC with 5 mM Keggin POMs (PW_12_, SiW_12_, H_2_W_12_) as a function of pH at 25°C. Top: Speciation diagram showing solution species as a function of pH. Labels: SiO_4_ for SiO_4_^4−^ and PO_4_ for PO_4_^3−^. (b) Illustration showing the mechanism of pH‐switching in hydrogels of CEs with superchaotropic Keggin POMs. (c–e) pH switchability in PW_12_/HPC solutions: (c) Zero‐shear viscosity (*η*_0_) from flow curves of 130 mM HPC with 5 mM PW_12_ as a function of pH. (d) SAXS profiles and (e) Raman spectra of 130 mM HPC with 5 mM PW_12_ at pH steps 1 to 3. Fits with Voigt profiles are shown as dashed lines. (f–h) pH switchability in PW_12_/MC hydrogel: (f) Chemical structure of methylcellulose. (g) Storage moduli (*G*′, solid circles) and loss moduli (*G*″, hollow circles) from frequency sweeps (*ω* = 10 rad s^−1^, *γ*% = 1%) of 130 mM MC with 2.5 mM PW_12_ as a function of pH. (h) Photographs showing inversion tests with “gel‐sol‐turbid gel‐sol” transition during consecutive pH steps.

The strong changes in solution structure upon POM speciation suggest that the flow properties of POM/HPC solutions can be tuned by pH. We therefore traced the rheological behavior of 130 mM HPC with 5 mM PW_12_, SiW_12_ and H_2_W_12_, as shown in Figure 4a. 130 mM HPC corresponds to five times the overlap concentration of HPC and represents an entangled polymer solution [77]. Zero‐shear viscosities were extracted by fitting the flow curves with a Carreau Yasuda model (Figure S28) and were correlated with speciation diagrams obtained from Raman spectroscopy. At low pH, superchaotropic PW_12_ acts as a physical crosslinker, increasing viscosity of 130 mM HPC from ∼0.02 Pa·s to ∼25 Pa·s. Upon addition of NaOH, the viscosity decreased slightly to ∼12 Pa·s and remained constant up to pH 5. Between pH 5 and 5.5, the conversion of plenary PW_12_ to lacunary PW_11_ is accompanied by a sharp decrease in the viscosity by three orders of magnitude. At higher pH, the viscosity remained constant and matched that of 130 mM HPC, consistent with the formation of non‐crosslinking lacunary PW_11_, tungstate, and phosphate ions. A similar pH tunable response was observed for SiW_12_, albeit with a relatively smaller decrease in viscosity by two orders of magnitude. Since H_2_W_12_ does not act as a physical crosslinker for HPC, no pH‐dependent viscosity changes were observed.

Additionally, we performed frequency sweeps to investigate the structural evolution of PW_12_/HPC solution during the pH speciation, as shown in Figure S29. At low pH, the sample exhibited a typical response for a viscoelastic liquid characterized by a *G*′ (storage modulus)—*G*″ (loss modulus) crossover. The crossover shifted to higher frequencies with increasing pH and eventually disappeared upon complete hydrolysis of PW_12_. We used the crossover frequency to calculate the crossover relaxation time (*τ*_crossover_), while the terminal relaxation time (*τ*_terminal_) was determined from flow curves, see Figure S31. A decreasing trend in the relaxation times suggested breakdown of large structures into smaller ones, consistent with the pH‐driven decrosslinking mechanism.

Consequently, the conversion of a physical crosslinking motif at low pH into a non‐crosslinking motif at high pH allowed us to develop pH‐switchable materials based on superchaotropic interactions (Figure 4b). Moreover, the base‐induced hydrolysis of Keggin‐type POMs is reversible via acidic polycondensation [78]. Hence, we tested the reversible switching of viscosity by adjusting the pH of PW_12_/HPC solution between pH 2 and pH 8 using NaOH and HCl. The zero‐shear viscosity of PW_12_/HPC as a function of pH along with corresponding SAXS and Raman spectra is shown in Figure 4c–e. The flow curves for the PW_12_/HPC solutions during the pH cycles are shown in Figure S32. By switching between low and high pH, the PW_12_/HPC solution reversibly transitioned between a viscoelastic liquid and a low‐viscosity solution, respectively, exhibiting three orders of magnitude change in the zero‐shear viscosity over three pH‐switching cycles. Raman spectra confirmed the reversible formation of PW_12_ after a base‐acid cycle. SAXS profiles revealed the recovery of the same intensity at high q and the disappearance of the correlation peak around *q* ≈ 0.6 nm^−1^ following a pH cycle, consistent with electrostatic screening due to salt (NaCl) formation during acid‐base neutralization. We performed similar pH switching experiments on HPC/SiW_12_ solutions and obtained comparable results (Figures S33 and S34).

To further demonstrate the generality of this pH‐switchable platform, we used a different cellulose ether polymer, methylcellulose (MC), see Figure 4f, to induce ‘gel‐to‐sol’ transitions. Figure 4g shows the pH‐switchable behavior for a hydrogel of 130 mM MC with 2.5 mM PW_12_, measured using frequency sweeps; see Figure S35. The storage modulus (G′) of the resulting hydrogel is in the order of 10^3^ Pa, consistent with the formation of a soft hydrogel network. When the pH was varied, we observed a sequence of macroscopic transitions from “*transparent gel → sol → turbid gel → sol*” as shown by inverted vial tests in Figure 4h. These transitions were accompanied by partially reversible changes in the storage modulus (*G*′) of roughly three to four orders of magnitude. Taken together, the above results show that the pH‐based switching of superchaotropic Keggin POMs can be used to impart pH‐responsivity to otherwise pH‐insensitive non‐ionic polymers.

## 3 Conclusion

In this work, we showed that superchaotropic binding of Keggin POMs with a non‐ionic cellulose ether, such as HPC, expands the pH stability range of superchaotropic plenary Keggin POMs. The binding to HPC leads to depletion of the POM from the solution and stabilizes the POM against base‐induced hydrolysis. The extent of the improvement in the POM's hydrolytic stability depends on the degree of superchaotropicity, which is determined by the charge density and hydration of the POM. For example, PW_12_ exhibited a stronger increase in pH stability than SiW_12_ due to its lower charge density, weaker hydration, and stronger binding, whereas H_2_W_12_, the plenary POM with the highest charge density, strongest hydration, and no ability to bind to HPC, showed no improvement in pH stability upon addition of HPC.

Increasing HPC concentration and thereby the concentration of free binding sites shifts the equilibrium toward bound POMs by Le Chatelier's principle and thus enhances the stabilizing effect. The stabilization by POM‐HPC binding was quantified as an increase in the formation constant of plenary superchaotropic POMs at the expense of non‐superchaotropic intermediate species that become suppressed.

We propose that superchaotropic binding to non‐ionic interfaces provides a general strategy to improve pH stability of POMs in water without the need for organic solvents or covalent/electrostatic modifications, enabling their applications in biological and aqueous systems at near neutral pH. Beyond the Keggin POMs investigated here, other pH‐sensitive superchaotropic POMs with diverse architectures, such as giant polyoxomolybdates [51], can be anticipated to be stabilized at non‐ionic interfaces as well. Such stability enhancement makes POM/soft matter hybrids viable for a broad range of catalytic, electronic, and pharmaceutical applications [1, 19]. The interplay between POM superchaotropicity and solution stability, therefore, should be considered as an important design criterion.

Furthermore, the pH‐induced conversion of plenary POMs into lacunary POMs and smaller anions allows for tunable transition between superchaotropic and kosmotropic behavior, allowing precise modulation of hydrogel structure of HPC from a crosslinked to a decrosslinked state. This establishes superchaotropic POMs as pH‐responsive crosslinkers, as evidenced by CP measurements, rheology, and SAXS/SANS.

We thus demonstrated that these POMs can be used to design pH‐switchable and reversibly tunable hydrogels, thereby expanding the available toolkit for physically crosslinked stimuli‐responsive materials. This pH‐responsive superchaotropicity is broadly transferable to non‐ionic soft matter interfaces—including micelles, surfactants, microgels [36], hydrotropes, polymers, and membranes—broadening the scope of responsive POM/soft matter hybrid materials. The simplicity of hydrogel fabrication, pH‐specific response, and the adaptability of this strategy to other biocompatible soft matter systems, combined with the built‐in catalytic/electronic properties of POM, suggests promising opportunities for the development of responsive materials for applications such as drug delivery, soft robotics, and sensors.

## Conflicts of Interest

The authors declare no conflicts of interest.

## Supporting information




**Supporting File 1**: Materials and Methods section: details on mass balance equations for speciation fits, details for SAXS/SANS fitting, and additional plots (pH titration, Raman, NMR, SAXS, SANS, rheology). The authors have cited additional references within the Supporting Information [79, 80, 81, 82, 83, 84, 85, 86, 87, 88, 89].

## Data Availability

The data that support the findings of this study are openly available in zenodo at https://doi.org/10.5281/zenodo.18153131.
