# Supplementary material for: Hydrogels Under Superchaotropic Control: Polyoxometalate Stabilization and pH‐Responsive Crosslinking in Cellulose Ether Solutions
Source: Angew Chem Int Ed Engl. 2026 Apr 16;65(22):e6958664. doi: 10.1002/anie.6958664 (PMC13206204; doi:10.1002/anie.6958664)
Supplement: Supplementary file 1 — Supporting File 1: Materials and Methods section: details on mass balance equations for speciation fits, details for SAXS/SANS fitting, and additional plots (pH titration, Raman, NMR, SAXS, SANS, rheology). The authors have cited additional references within the Supporting Information [79, 80, 81, 82, 83, 84, 85, 86, 87, 88, 89]. [file ANIE-65-e6958664-s001.pdf]

Supplementary Information

Hydrogels Under Superchaotropic Control: Polyoxometalate Stabilization and pH-Responsive Crosslinking in Cellulose Ether Solutions

Vighnesh B. Lokare[a], Amina Ledinic[a], Nina Wehr[b] and Max Hohenschutz*[a]

[a] V. B. Lokare, A. Ledinic, Dr. M. Hohenschutz
Institute of Physical Chemistry
RWTH Aachen University
Landoltweg 2, 52072 Aachen (Germany)
E-mail: M. Hohenschutz: hohenschutz@pc.rwth-aachen.de

[b] N. Wehr
Institute of Technical and Macromolecular Chemistry
RWTH Aachen University
Worringerweg 2, 52074 Aachen (Germany)

Table of Contents

S1. Materials and Methods 3
S2. pH Titration Profiles 5
S3. Raman Spectroscopic Measurements 7
S3.1. Raman spectra with Voigt profiles 7
S3.2. Band positions in Raman spectra 16
S3.3. Relative concentration from Raman spectra 25
S3.4. Modelling Speciation of POMs 26
S3.5. pH Speciation in H_2O and D_2O studied using Raman spectroscopy 32
S3.6. Speciation in two phases 33
S4. ^{1}H NMR Measurements 34
S5. ^{31}P NMR Measurements 35
S6. Solvation free energies of POM species 37
S7. Modeling of the SAXS and SANS profiles 38
S7.1. Fitting of SANS profiles 39
S7.2. Fitting of SAXS profiles for POM species in water 40
S7.3. Fitting of SAXS profiles 41
S7.4. SAXS profile for HPC/PW_{12} 44
S7.5. SANS and SAXS profile for HPC/SiW_{12} 46
S7.6. PW_{11} as non-interacting species in solution 46
S8. Coupled equilibria 47
S9. Rheology 47
S10. pH Responsive Solution and Hydrogel 50
S11. References 52

S1. Materials and Methods

Materials:

The α-Keggin polyoxometalates (POM) phosphotungstic acid ($H_3[PW_{12}O_{40}].xH_2O$, PW_{12}, M_w=3267.05 g/mol, p>99.9%), silicotungstic acid ($H_4[SiW_{12}O_{40}].xH_2O$, SiW_{12}, M_w=3265.17 g/mol, p>99.9%) and ammonium metatungstate, ($(NH_4)_6[H_2W_{12}O_{40}]\cdot yH_2O$, H_2W_{12}, M_w=3037.3 g/mol p>99.9%) were purchased from Sigma Aldrich, with numbers of hydration water x=21.5 and y=4.5 determined by thermogravimetric analysis. The cellulose ethers (CEs) - hydroxypropyl cellulose (HPC, average M_w=88 kDa, N=230, moles of substitution of hydroxypropyl units = 3.8)[1] was obtained from Nisso and methyl cellulose (MC A15LV, M_w=20-380 kDa, M_v= 42 kDa, degrees of substitution of methyl groups = 1.74)[2] was obtained from J. Rattenmaier & Söhne GmbH & CoKG. Sodium tungstate dihydrate ($Na_2WO_4.2H_2O$), trimethylsilylpropanesulfonate (DSS, p>97%), and phosphoric acid (H_3PO_4. 8130 mM in H_2O) was purchased from Sigma Aldrich. D_2O (p>99.9%) and NaOD (40% in D_2O) were purchased from Deutero. NaOH (1 M) and HCl (1 M) were obtained from Carl Roth. HPC and MC were dialyzed against Milli-Q water (σ = 0.055 µS/cm) using a dialysis tubing (molecular weight cut-off 12-14 kDa). All solutions were prepared using double-distilled Milli-Q water. $H_3[PW_{12}O_{40}]$, was purified by ether extraction to remove phosphate impurity following the procedure reported by Matijević et al.[3] All other chemicals were used without further purification.

Sample preparation:

All solutions were prepared using double-distilled Milli-Q H_2O. Each sample was prepared from a stock solution of Keggin POM (PW_{12}, SiW_{12} or H_2W_{12}), HPC and NaOH, mixed in required proportions to make 2 mL solutions. The concentration of Keggin POMs in all final solutions was maintained at 5 mM. For each POM, three types of solutions were prepared: POM-only solution (in water, without HPC), 26 mM HPC and 130 mM HPC. Concentrations of HPC (and MC) are expressed as molarity of the monomer throughout the paper. NaOH was added in molar equivalents relative to the POM concentration. The superchaotropic polymer/POM mixtures were prepared by adding the required amount of pre-dissolved polymer solution to a POM solution followed by addition of NaOH. Highly viscous and gelled samples were subjected to a single heating cycle of 60°C for 15 min in a stirred water bath for homogenization. Some sample series were also prepared using D_2O and NaOD, instead of H_2O and NaOH, as required for specific measurements. All samples were measured only after pH equilibrium had been established. All measurements were performed at 25°C unless stated otherwise. Samples containing 130 mM MC were prepared by following the same method as for HPC.

pH measurements:

pH measurements were performed on a Metrohm 744 pH Meter with a Biotrode electrode. The pH-electrode was calibrated with buffers of pH 4 and pH 10. pH was measured immediately after sample preparation and periodically over the subsequent 24 hours, until equilibrium was reached and stable readings were observed. All pH values reported indicate $pH_{measured}$ (i.e. pH reading of the calibrated electrode). For all samples in D_2O, their pD value can be estimated by the using the relation; $pD = pH_{measured} + 0.41$

Raman spectroscopy:

The Raman spectra were obtained using the Kaiser Optical Systems RAMAN RXN2 analyzer. The probe has a laser excitation line of 780 nm with a constant power of 150 mW, exposure time is 5.00 s and a repetition rate of 10. The spectral range is from 900 cm^{-1} to 1050 cm^{-1}.

The Raman spectra were analyzed using Fityk software.[4] A linear baseline was defined by drawing a line through the spectral minima to correct for the background contributions. The corrected spectra were deconvoluted by fitting with a sum of Voigt profiles giving characteristic band positions and areas. Raman band positions serve to identify the species. The area under the band gives the absolute/relative abundance of each species in the solution as shown in Section S3.3.

The speciation of POMs was modeled using HySS2009 (Hyperquad Simulation and Speciation) software using formation reactions discussed in Section S3.4.[5]

Cloud point measurements:

The cloud point temperature (CP) of the solutions was determined by visual observation. The samples were heated in a stirred water bath at a controlled rate of 2 °C per minute, monitored using a precision thermometer. The CP was recorded at the first observable appearance of turbidity, which indicated an onset of phase separation. To improve visibility, observations were made against a black background. Due to the subjective nature of visual detection, the measurement error was estimated to be ±1 °C.

Rotational rheology:

Rheological measurements were conducted on a stress controlled DHR-3 rheometer from TA instruments with a 40mm 1.008° cone-plate geometry at 25°C. To prevent water evaporation, 0.05 Pas silicone oil was employed as a solvent trap. The steady shear viscosity was measured over shear rates from 0.01 s^{-1} to 1000 s^{-1}. Amplitude sweeps were carried out to determine the linear viscoelastic region (LVER) at a constant frequency of 10 rad s^{-1}, while varying the strain from 0.1% to 100%. All frequency sweeps were performed within the LVER at a constant strain, with frequency varied between 100 rad s^{-1} to 0.1 rad s^{-1}. Only data points with torque (τ) > 1 μNm were utilized above the detection limit of the transducer.

Small Angle Neutron Scattering (SANS):

SANS measurements were performed on the Larmor instrument at the ISIS Neutron & Muon Source (Rutherford Appleton Laboratory, Didcot, UK). The instrument operates in time-of-flight mode using a white beam of neutron having wavelength in the range of 0.9 - 13.0 Å. The sample to detector distance was set to 4 m, covering an effective q-range of 0.005 - 0.67 $Å^{-1}$. Round quartz cuvettes from Hellma with pathlengths of 1 mm served as sample containers. All samples were prepared with D_2O instead of H_2O. Measurements were performed at 25°C using a thermostated sample holder. Data reduction was performed on the raw intensity by correcting for background scattering and subtracting the scattering contributions from the solvent and cuvette.

Small Angle X-ray Scattering (SAXS):

SAXS measurements using Cu radiation (λ = 0.154 nm) were performed at the Institute of Technical and Macromolecular Chemistry (ITMC) at RWTH Aachen on a Nano-inXider system from Xenocs equipped with simultaneous SAXS/WAXS detection. The accessible q range was 0.01 nm^{-1} to 10 nm^{-1}. The instrument used a Dectris Pilatus 3 hybrid photon counting detector to record the scattered beam at a sample to detector distance (SDD) of 938 mm. The 2D detector intensities were azimuthally averaged, and reduced using detector noise, sample transmission and sample thickness. Scattering contributions from the empty capillary was subtracted from the raw intensity. Sealed 1.5 mm (approx.) borosilicate capillaries from WJM Glass Müller GmbH served as sample containers. Exact sample thickness was measures with a micrometer.

^{1}H Nuclear magnetic resonance measurements:

^{1}H NMR measurements were recorded using an Avance400 (Bruker, Billerica, MA, USA) spectrometer with 128 scans per measurement. Sodium trimethylsilylpropanesulfonate (DSS) was used as an external calibration standard. Samples were prepared using D_2O as solvent. Chemical shifts are reported in parts per million (ppm).

^{31}P Nuclear magnetic resonance measurements:

Solution ^{31}P NMR were recorded using an Avance400 (Bruker, Billerica, MA, USA) spectrometer. 8130 mM H_3PO_4 was used as an external calibration standard in a coaxial inset that was set into the NMR tube. Chemical shifts are reported with respect to 8130 mM H_3PO_4 in parts per million (ppm), with all chemical shifts downfield of the reference reported as positive values.

For the solid-state NMR experiments the samples were sedimented into the NMR rotor for 2 h with a g-force of 200,000 g, using home-built tools and a Beckman Coulter Optima XPN 80 centrifuge. The solid-state NMR experiments were performed at a Bruker Avance III HD spectrometer with a static magnetic field strength of 11.7 T. All experiments were recorded with a 3.2 mm triple resonance Bruker Biospin probe at a MAS frequency of 17 kHz.

S2. pH Titration Profiles

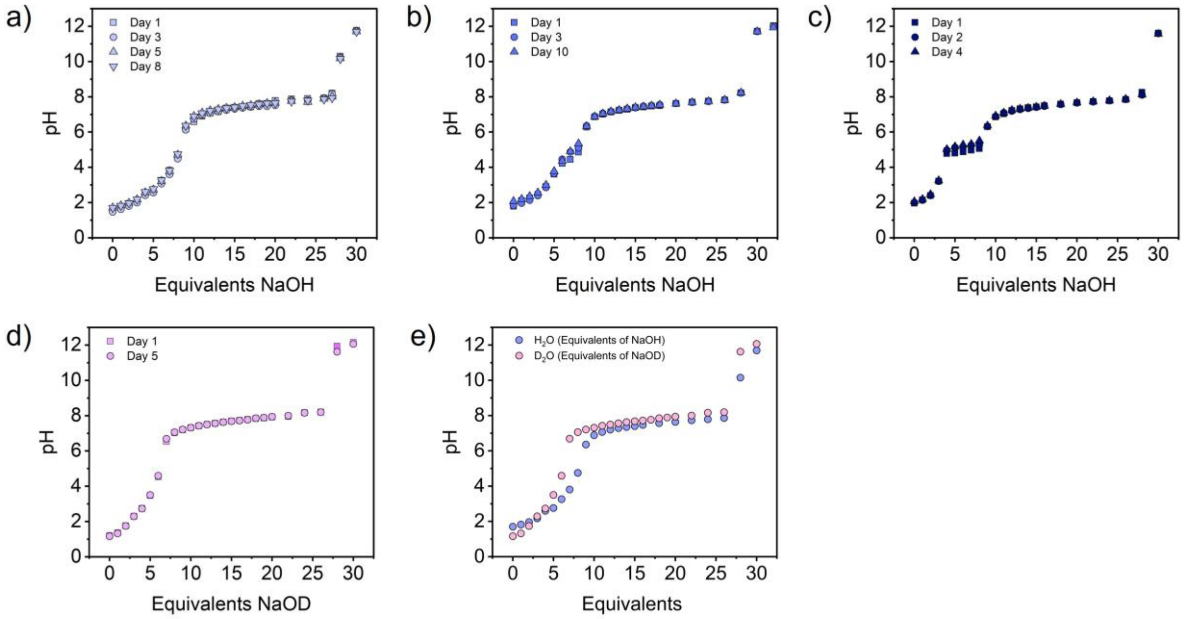

Figure S1. pH titration curves of 5 mM PW_{12} in a) H_2O, b) 26 mM HPC, c) 130 mM HPC with molar equivalents of NaOH and d) in D_2O with equivalents of NaOD over several days. e) Comparison between pH titration profile of 5 mM PW_{12} in H_2O and D_2O NaOH or NaOD were added in molar equivalents relative to the POM concentration.

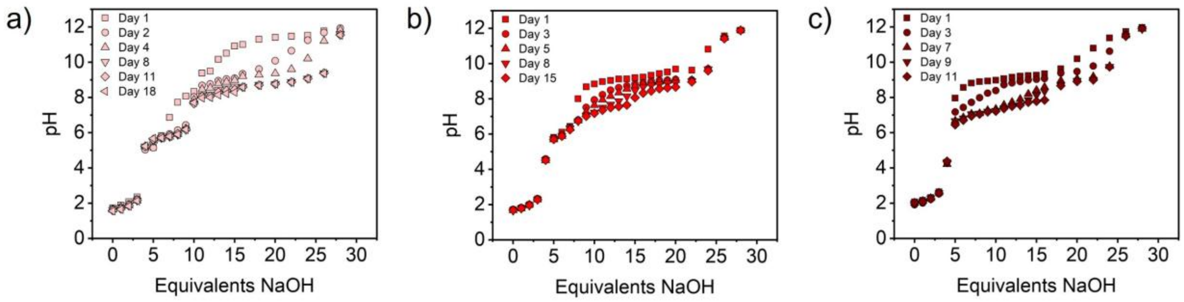

Figure S2. pH titration curves of 5 mM SiW_{12} in a) H_2O, b) 26 mM HPC, and c) 130 mM HPC with molar equivalents of NaOH over several days.

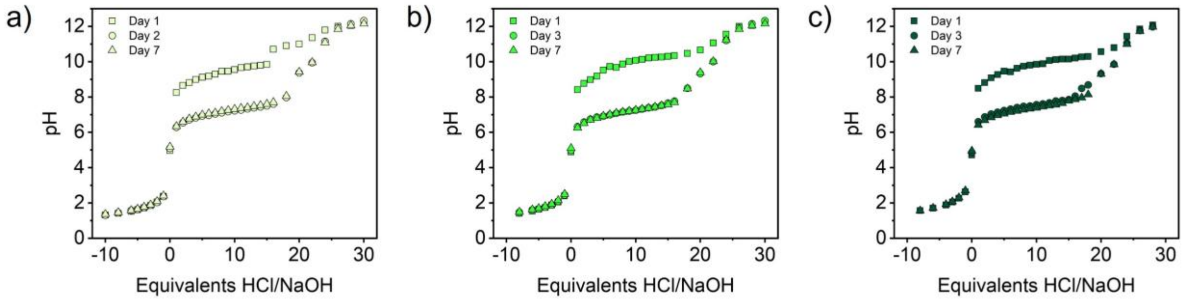

Figure S3. pH titration curves of 5 mM H_2W_{12} in a) H_2O, b) 26 mM HPC, and c) 130 mM HPC with molar equivalents of NaOH over several days.

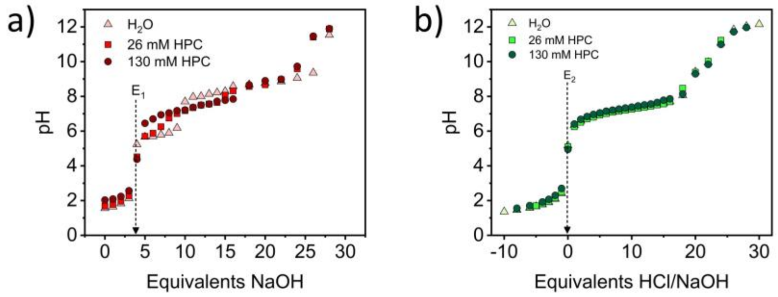

Figure S4. pH titration curves of 5 mM Keggin-type POMs a) SiW_{12} and b) H_2W_{12} in H_2O, in presence of 26 mM HPC and 130 mM HPC. pH adjusted for SiW_{12} using NaOH and for H_2W_{12} using HCl (negative axis) and NaOH (positive axis). NaOH and HCl were added in molar equivalents relative to the POM concentration. SiW_{12} shows an equivalence point at four equivalents NaOH corresponding to the neutralization of all four acidic protons. Addition of HPC raises the reached pH after equivalence and indicates a later onset of hydrolysis of SiW_{12}. By comparison, H_2W_{12} shows an apparent equivalence point at 0 equivalents, which reflects its lack of acidic protons, since it is used in its ammonium form.

S3. Raman Spectroscopic Measurements

S3.1. Raman spectra with Voigt profiles

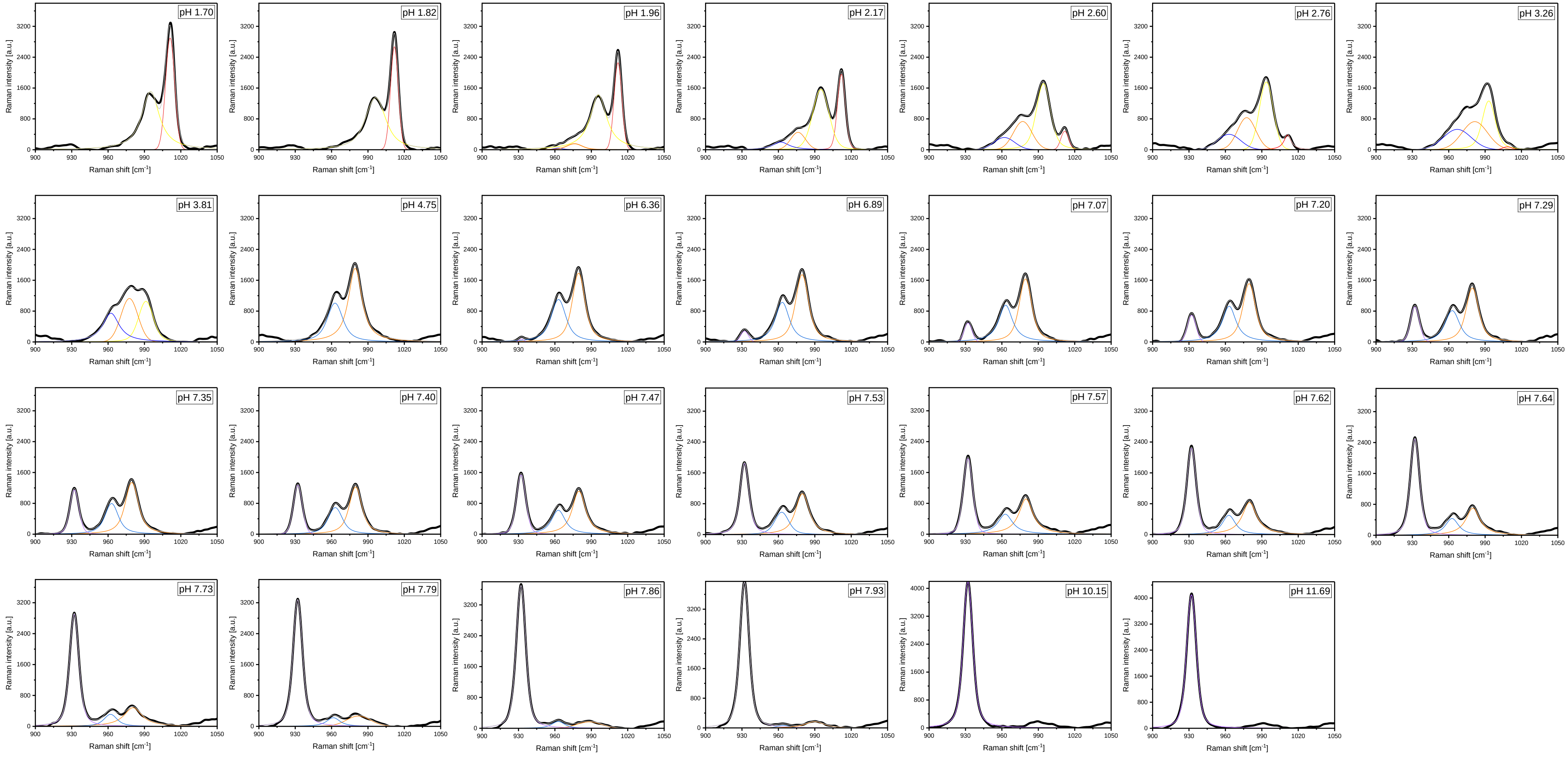

Figure S5. Raman spectra for 5 mM PW_{12} in H_2O at different pH values.

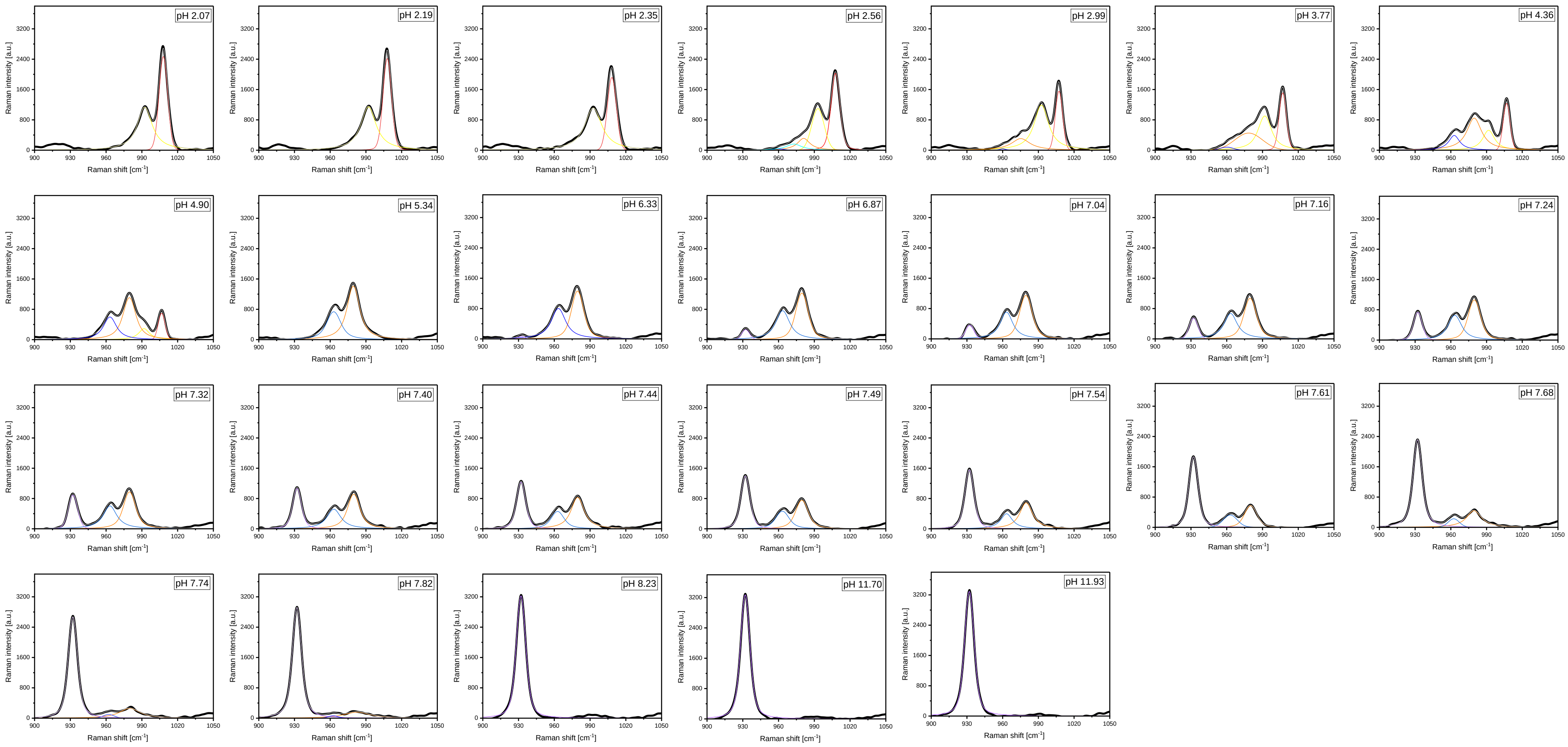

Figure S6. Raman spectra for 5 mM PW_{12} in presence of 26 mM HPC at different pH values.

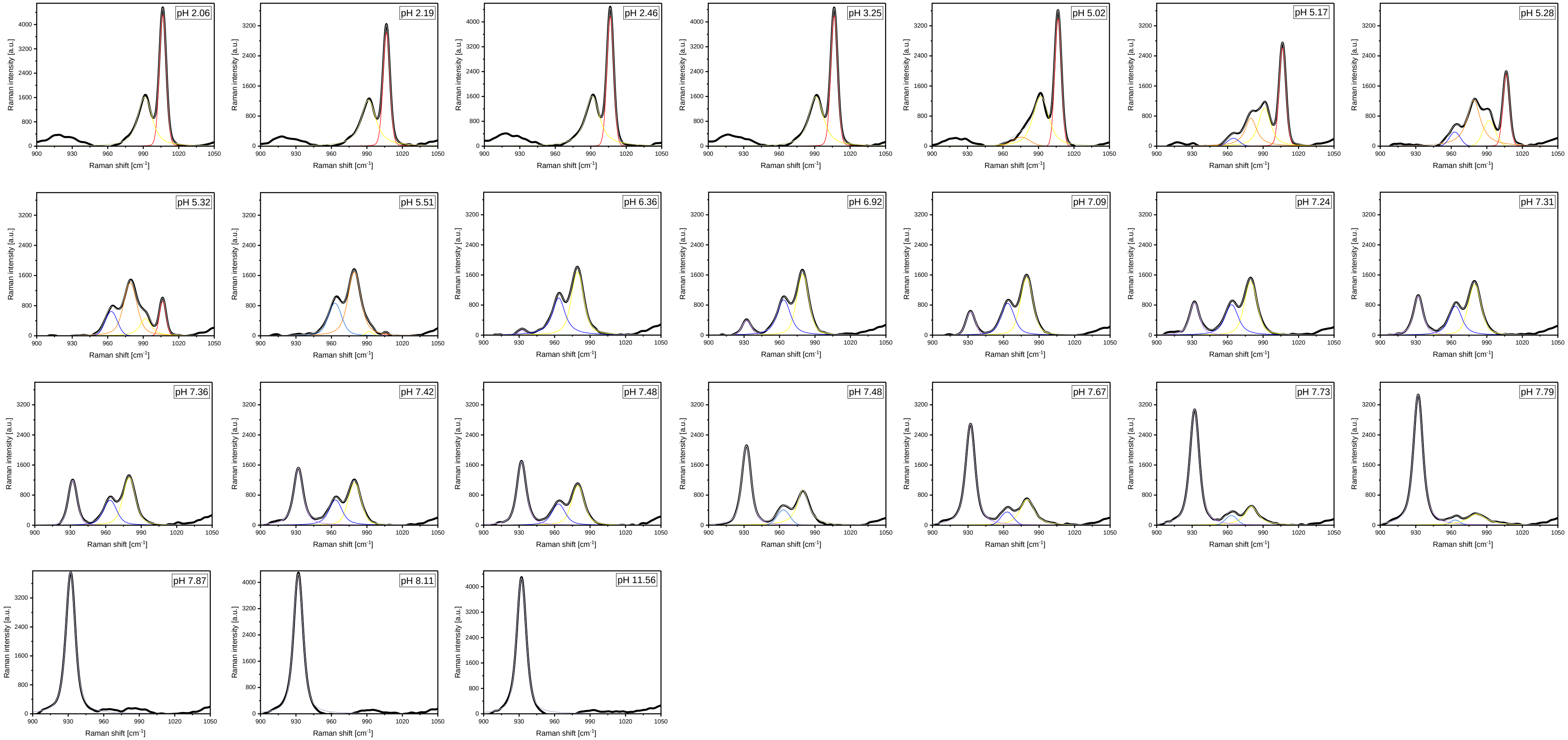

Figure S7 Raman spectra for 5 mM PW_{12} in presence of 130 mM HPC at different pH values.

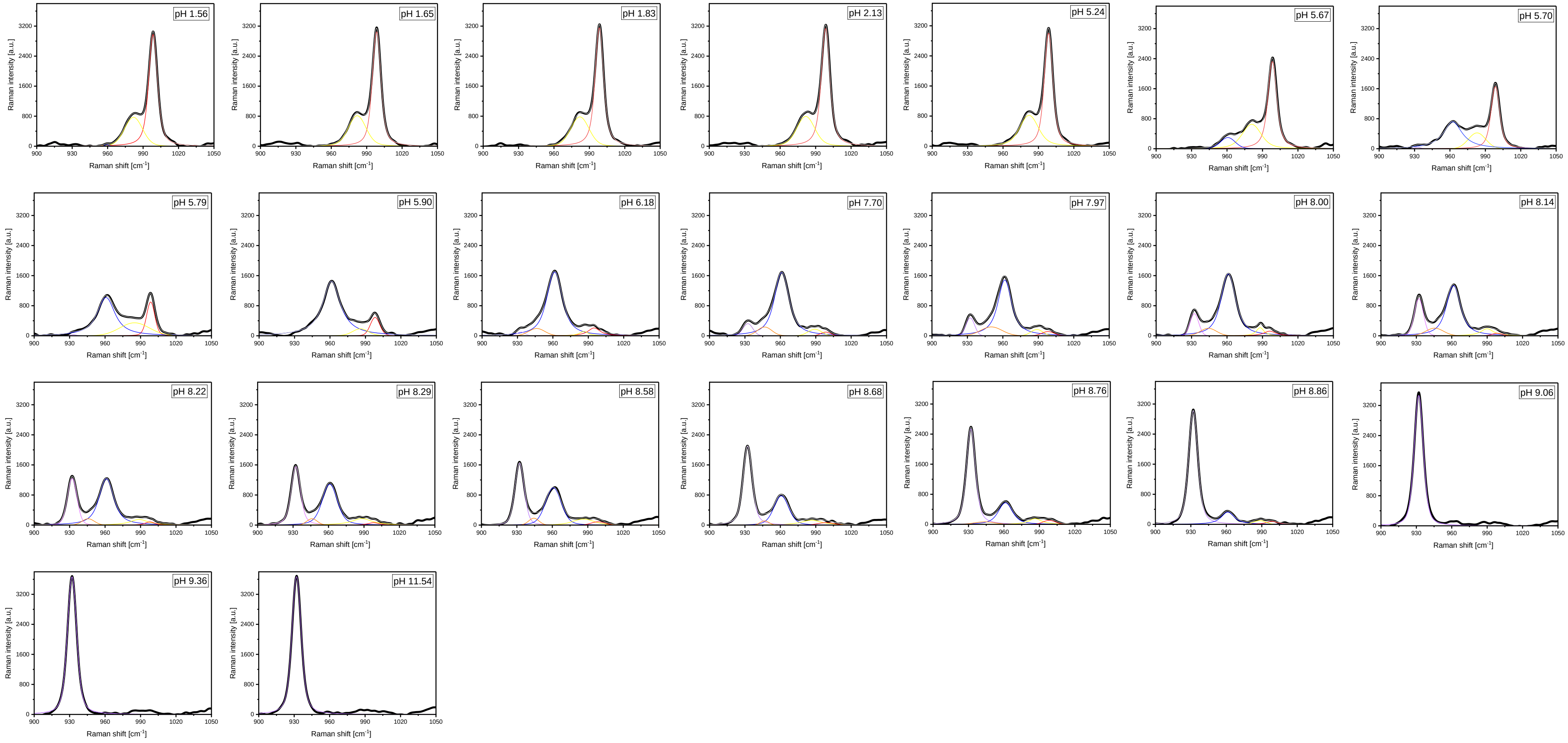

Figure S8. Raman spectra for 5 mM SiW_{12} in H_2O at different pH values.

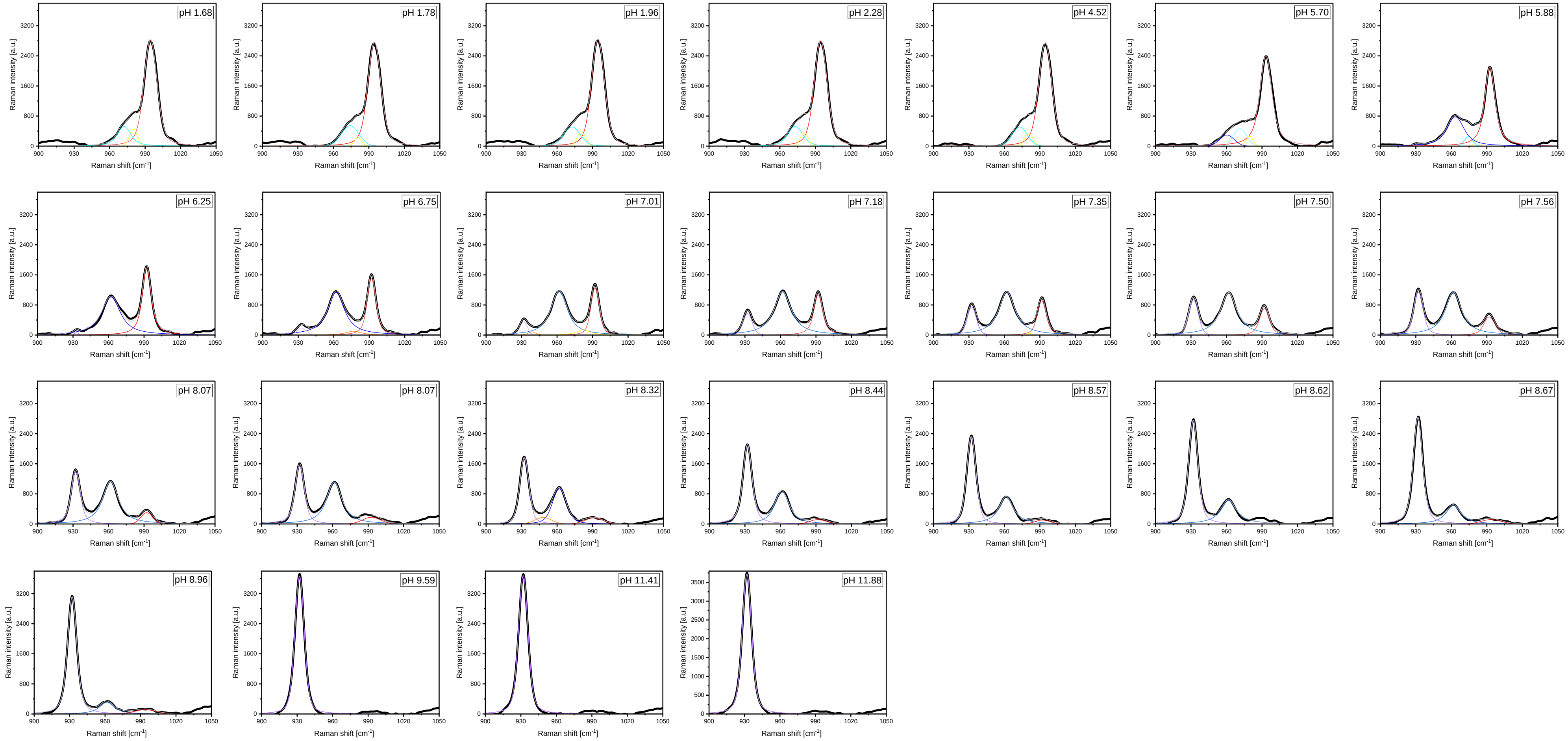

Figure S9. Raman spectra for 5 mM SiW_{12} in presence of 26 mM HPC at different pH values.

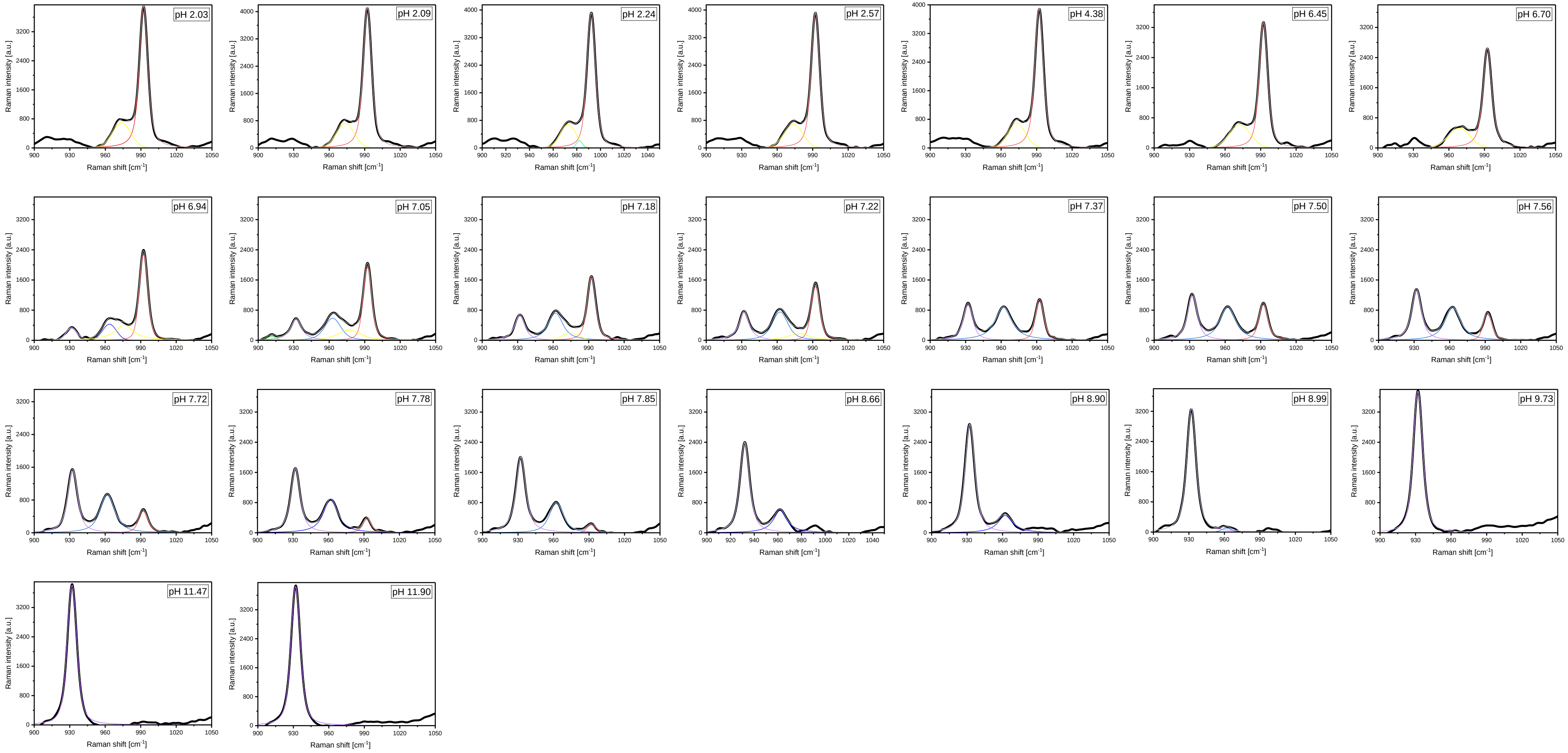

Figure S10. Raman spectra for 5 mM SiW_{12} in presence of 130 mM HPC at different pH values.

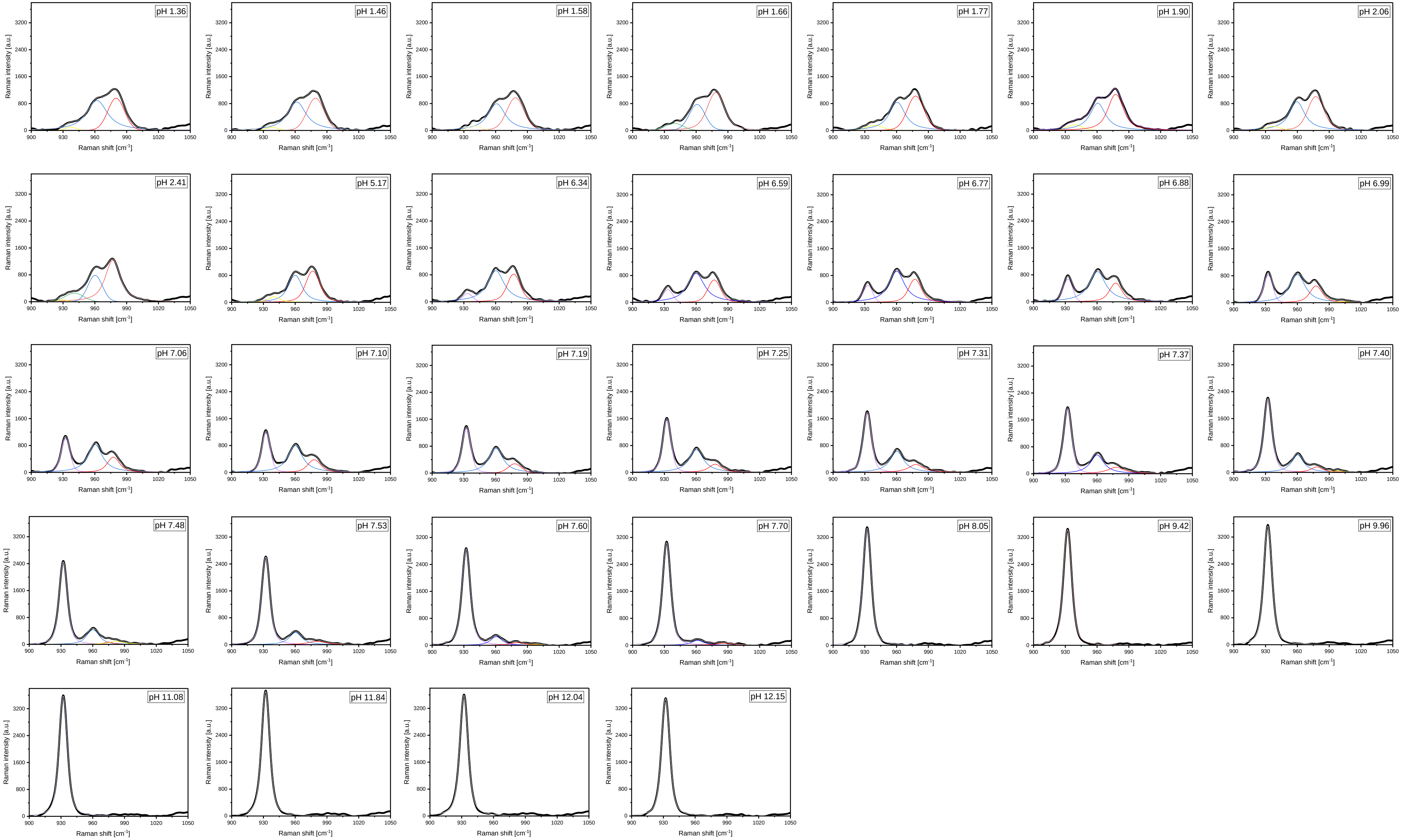

Figure S11. Raman spectra for 5 mM H_2W_{12} in H_2O at different pH values.

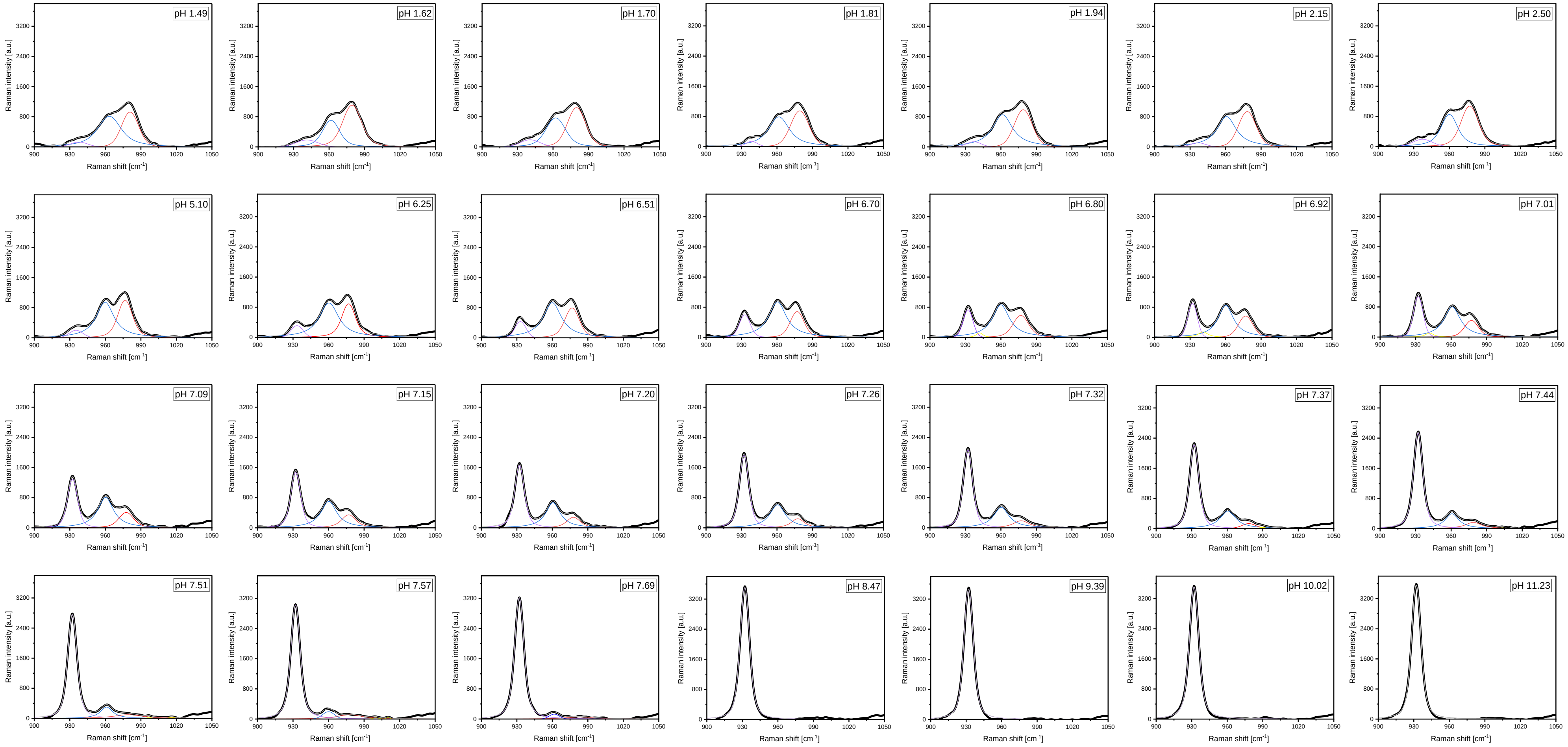

Figure S12. Raman spectra for 5 mM H_2W_{12} in presence of 26 mM HPC at different pH values.

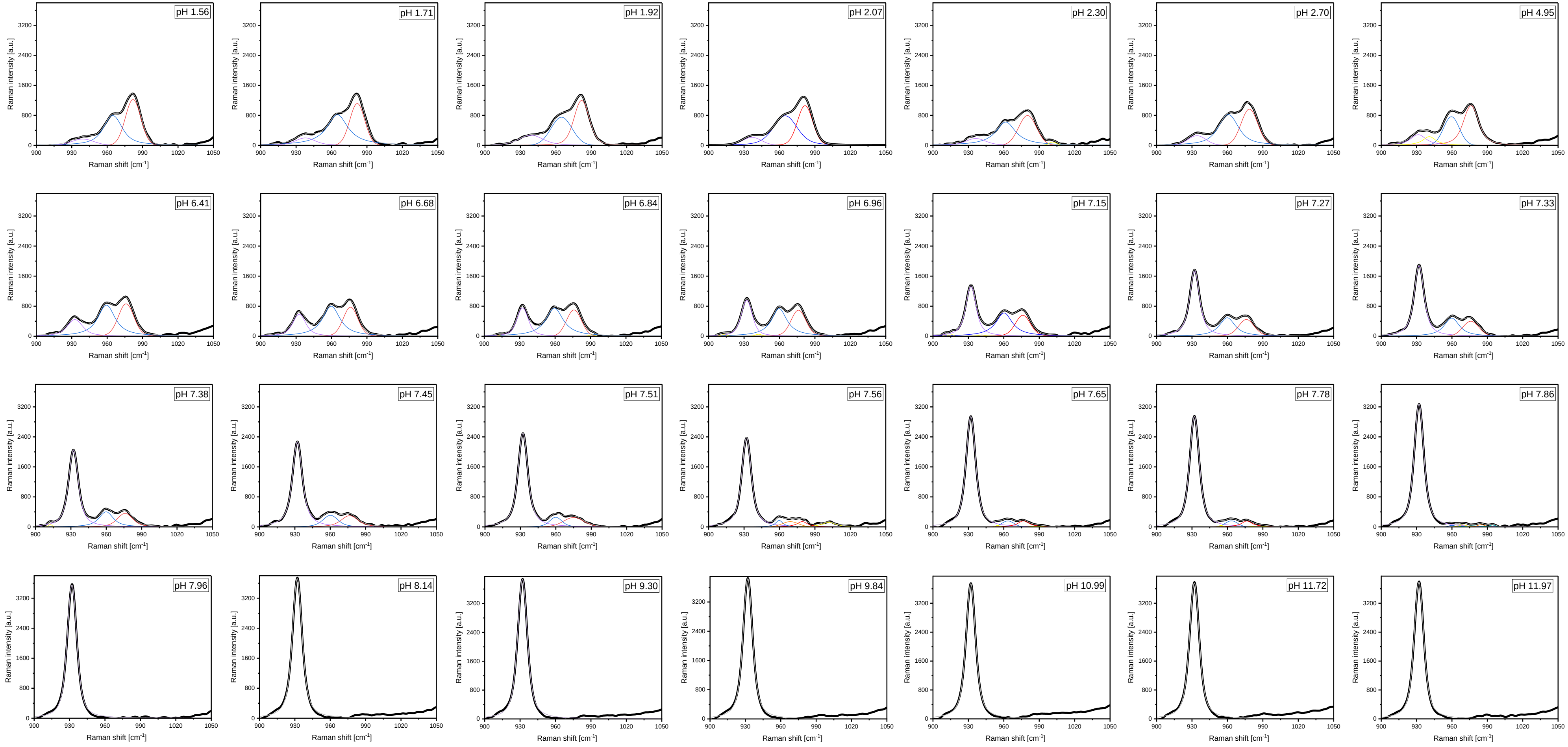

Figure S13. Raman spectra for 5 mM H_2W_{12} in presence of 130 mM HPC at different pH values.

S3.2. Band positions in Raman spectra

Table S1. Raman band positions for 5 mM PW_{12} in H_2O at different pH values, obtained from Voigt profile fitting of the Raman spectra.

pH	Band 1 $[PW_{12}O_{40}]^{3-}$	Band 2 $[PW_{12}O_{40}]^{3-}$	Raman shift [cm^{-1}] Band 3 Not assigned	Band 4 $[PW_{11}O_{39}]^{7-}$	Band 5 WO_4^{2-}
1.7	1011.2	995.0	974.9		
1.82	1011.8	996.2		960.0	
1.96	1011.9	995.8	976.1	962.7	
2.17	1011.9	995.3	977.5	963.9	
2.6	1011.7	994.2	977.2	962.0	
2.76	1011.2	993.7	977.5	963.0	
3.26	1008.4	993.0	981.5	967.0	
3.81		992.0	978.7	963.0	
4.75			979.5	962.8	
6.36			979.5	963.0	932.3
6.89			979.5	963.2	932.1
7.07			979.4	963.1	932.1
7.2			979.5	963.1	932.2
7.29			979.5	962.9	932.1
7.35			979.6	962.9	932.1
7.4			979.8	963.0	932.1
7.47			979.8	962.8	932.1
7.53			979.8	962.7	932.0
7.57			979.8	962.8	932.0
7.62			979.8	962.8	932.0
7.64			979.9	962.7	932.1
7.73			980.5	962.0	932.1
7.79			982.2	961.4	932.1
7.86			988.2	962.7	932.0
7.93			989.9	963.9	932.0
10.15					932.0
11.69					932.1

Table S2. Raman band positions for 5 mM PW_{12} in presence of 26 mM HPC at different pH values, obtained from Voigt profile fitting of the Raman spectra.

	Raman shift [cm^{-1}]				
**pH**	**Band 1** **$[PW_{12}O_{40}]^{3-}$**	**Band 2** **$[PW_{12}O_{40}]^{3-}$**	**Band 3** **Not assigned**	**Band 4** **$[PW_{11}O_{39}]^{7-}$**	**Band 5** **WO_4^{2-}**
2.07	1008.0	992.4			
2.19	1007.9	992.1			
2.35	1008.1	993.1	981.5		
2.56	1007.8	993.0	972.8	960.0	
2.99	1007.3	992.5	975.2	960.0	
3.77	1007.0	991.9	978.4	960.0	
4.36	1006.8	991.7	979.6	962.8	
4.9	1006.6	991.8	979.5	963.2	
5.34			979.4	963.0	
6.33			979.5	963.4	932.1
6.87			979.6	963.5	932.4
7.04			979.5	963.2	932.4
7.16			979.6	963.3	932.2
7.24			979.7	963.3	932.2
7.32			979.6	963.3	932.2
7.4			979.8	963.1	932.1
7.44			979.6	962.8	932.1
7.49			979.5	963.2	932.1
7.54			979.7	963.3	932.1
7.61			980.1	963.0	932.1
7.68			979.4	962.4	932.1
7.74			979.8	962.1	932.1
7.82			982.6	962.5	932.1
8.23					932.1
11.70					932.1
11.93					932.0

Table S3. Raman band positions for 5 mM PW_{12} in presence of 130 mM HPC at different pH values, obtained from Voigt profile fitting of the Raman spectra.

pH	Raman shift [cm^{-1}] Band 1 $[PW_{12}O_{40}]^{3-}$	Band 2 $[PW_{12}O_{40}]^{3-}$	Band 3 Not assigned	Band 4 $[PW_{11}O_{39}]^{7-}$	Band 5 WO_4^{2-}
2.06	1006.6	991.3			
2.19	1006.6	991.3			
2.46	1006.6	991.4			
3.25	1006.6	991.2			
5.02	1006.6	992.0	975.2		
5.17	1006.5	991.2	979.7	964.9	
5.28	1006.5	992.0	979.0	963.0	
5.32	1006.4	992.0	979.0	963.0	
5.51	1006.1	992.0	979.4	963.0	
6.36			979.4	963.5	932.2
6.92			979.6	963.6	932.2
7.09			979.6	963.5	932.1
7.24			979.5	963.3	931.9
7.31			979.7	963.5	932.1
7.36			979.9	963.7	932.0
7.42			979.6	963.4	932.0
7.48			979.7	963.3	932.0
7.58			979.8	963.3	932.0
7.67			980.0	963.0	932.0
7.73			980.1	963.1	932.0
7.79				963.3	931.9
7.87					932.0
8.11					932.0
11.56					932.0

Table S4. Raman band positions for 5 mM SiW_{12} in H_2O at different pH values, obtained from Voigt profile fitting of the Raman spectra.

pH	Raman shift [cm^{-1}]<br>Band 1<br>$[SiW_{12}O_{40}]^{4-}$	Band 2<br>$[SiW_{12}O_{40}]^{4-}$	Band 3<br>$[SiW_{11}O_{39}]^{8-}$	Band 4<br>Not assigned	Band 5<br>WO_4^{2-}
1.56	998.5	981.9	959.5		
1.65	998.5	982.0			
1.83	998.5	981.9			
2.13	998.5	981.6			
5.24	998.6	982.0			
5.67	998.4	980.8	960.7		
5.7	998.5	982.9	962.3		932.6
5.79	998.6	984.9	960.8		933.0
5.9	998.1	985.7	961.4		
6.18	994.9	986.2	961.5	946.3	933.0
7.7	998.5	989.2	961.2	946.6	932.5
7.97	998.2	988.7	961.6	950.3	932.4
8	996.3	988.1	961.1	944.2	932.4
8.14	996.2	988.0	961.1	944.2	932.4
8.22	998.0	988.7	961.1	945.7	932.1
8.29	998.1	988.2	961.0	946.0	932.3
8.58	998.8	986.3	960.9	944.3	932.3
8.68	998.8	986.4	960.8	946.6	932.2
8.76	998.4	986.3	961.3	943.9	932.0
8.86	998.7	988.3	961.2		932.0
9.06					932.0
9.36					932.0
11.54					932.0

Table S5. Raman band positions for 5 mM SiW_{12} in presence of 26 mM HPC at different pH values, obtained from Voigt profile fitting of the Raman spectra.

pH	Raman shift [cm^{-1}]				
	Band 1 $[SiW_{12}O_{40}]^{4-}$	Band 2 $[SiW_{12}O_{40}]^{4-}$	Band 3 Not assigned	Band 4 $[SiW_{11}O_{39}]^{8-}$	Band 5 WO_4^{2-}
1.68	994.6	980.1	972.2		
1.78	994.7	980.8	974.2		
1.96	994.6	980.5	972.7		
2.28	994.5	980.5	972.8		
4.52	994.7	980.7	973.0		
5.7	993.3	979.5	971.5	960.3	
5.88	992.6	981.0	974.5	963.4	930.6
6.25	992.3			962.0	933.0
6.75	992.0		976.6	962.0	933.1
7.01	992.1	982.0		961.7	931.8
7.18	991.9			961.8	932.3
7.35	991.6	981.3		962.0	932.4
7.5	992.0			961.8	932.1
7.56	992.1			961.7	932.3
7.65	992.3			961.7	932.3
8.07	992.9			961.4	932.1
8.32	992.3			961.9	932.3
8.44	991.8			961.2	932.1
8.57	992.4			961.5	932.0
8.62				961.8	931.9
8.67	993.7			961.7	931.8
8.96	994.1			961.6	932.1
9.59					932.1
11.41					932.0
11.88					932.1

Table S6. Raman peak positions for 5 mM SiW_{12} in presence of 130 mM HPC at different pH values, obtained from Voigt profile fitting of the Raman spectra.

pH	Band 1 $[SiW_{12}O_{40}]^{4-}$	Band 2 $[SiW_{12}O_{40}]^{4-}$	Raman shift [cm^{-1}] Band 3 Not assigned	Band 4 $[SiW_{11}O_{39}]^{8-}$	Band 5 WO_4^{2-}
2.03	992.3		973.6		
2.09	992.3		973.6		
2.24	992.2	982.1	972.7		
2.57	992.3		973.7		
4.38	992.2		973.2		
6.45	992.4		972.4		
6.7	992.5		968.7		
6.94	992.5		976.1	963.6	931.5
7.05	992.4		977.1	963.0	932.0
7.18	992.4		974.8	962.0	931.8
7.22	992.4		978.4	962.4	932.0
7.37	992.5			962.1	932.0
7.5	992.3			962.2	932.3
7.56	992.1			962.5	932.0
7.72	992.2			961.8	932.4
7.78	990.1			961.6	932.3
7.85	991.9			962.3	932.3
8.66				961.8	932.0
8.9				962.2	932.3
8.99				961.7	932.0
9.73					932.0
11.47					932.0
11.9					932.0

Table S7. Raman band positions for 5 mM H_2W_{12} in H_2O at different pH values, obtained from Voigt profile fitting of the Raman spectra.

	Raman shift [cm^{-1}]			
**pH**	**Band 1** $[H_2W_{12}O_{40}]^{6-}$	**Band 2** $[H_2W_{12}O_{40}]^{6-}$	**Band 3** Not assigned	**Band 4** WO_4^{2-}
1.36	980.0	962.0	937.2	
1.46	979.2	961.5	938.1	
1.58	978.7	960.9	939.8	
1.66	978.5	960.9	939.2	
1.77	978.0	960.5	939.3	
1.9	977.6	960.6	939.1	
2.06	977.4	959.6	937.5	
2.41	977.1	960.2	938.5	
5.17	976.6	959.9	938.1	
6.34	977.0	960.2		933.5
6.59	977.0	959.9		933.3
6.77	977.2	960.0	941.5	932.5
6.88	977.6	960.4		932.5
6.99	977.6	960.0		932.6
7.06	977.6	960.4		932.4
7.1	977.9	960.1		932.4
7.19	978.0	960.3		932.3
7.25	977.9	960.2		932.3
7.31	978.3	960.2		932.3
7.37	977.9	960.0		932.2
7.4	978.3	960.2		932.2
7.48	982.6	960.2		932.2
7.53	980.5	960.3		932.2
7.6	980.8	960.6		932.1
7.7	986.2	961.4		932.1
8.05				932.1
9.42				932.1
9.96				932.1
11.08				932.1
11.84				932.1
12.04				932.1
12.15				932.1

Table S8. Raman band positions for 5 mM H_2W_{12} in presence of 26 mM HPC at different pH values, obtained from Voigt profile fitting of the Raman spectra.

	**Raman shift [cm^{-1}]**			
**pH**	**Band 1 $[H_2W_{12}O_{40}]^{6-}$**	**Band 2 $[H_2W_{12}O_{40}]^{6-}$**	**Band 3 Not assigned**	**Band 4 WO_4^{2-}**
1.49	980.9	963.4	937.7	
1.62	979.4	961.6	941.3	
1.7	979.8	962.2	940.7	
1.81	979.2	961.6	938.5	
1.94	978.9	961.4	937.1	
2.15	978.3	960.8	936.9	
2.5	977.2	960.0	937.4	
5.1	976.7	959.8	935.3	
6.25	976.9	960.4		933.4
6.51	976.7	960.0		933.0
6.7	977.0	960.4		932.8
6.8	976.8	960.0	942.0	932.2
6.92	977.0	960.1	941.0	932.1
7.01	977.1	960.8	941.8	932.2
7.09	977.6	960.2		932.3
7.15	977.0	960.2		932.3
7.2	977.8	960.5		932.2
7.26	977.6	960.4		932.2
7.32	977.1	960.3		932.1
7.37	978.5	960.5		932.1
7.44	978.8	960.7		932.2
7.51	979.3	961.0		932.1
7.57	978.1	960.0		932.1
7.69	983.3	961.6		932.1
8.47				932.1
9.39				932.1
10.02				932.0
11.23				932.1

Table S9. Raman band positions for 5 mM H_2W_{12} in presence of 130 mM HPC at different pH values, obtained from Voigt profile fitting of the Raman spectra.

	Raman shift [cm^{-1}]			
pH	Band 1 $[H_2W_{12}O_{40}]^{6-}$	Band 2 $[H_2W_{12}O_{40}]^{6-}$	Band 3 Not assigned	Band 4 WO_4^{2-}
1.56	981.9	964.8	939.4	
1.71	976.1	960.2	941.4	
1.92	981.8	964.8	938.9	
2.07	981.7	964.8	936.7	
2.3	980.1	961.7	936.3	
2.7	978.6	960.8	934.2	
4.95	976.2	959.6	940.8	931.5
6.41	976.0	959.4		932.7
6.68	976.1	959.8		932.7
6.84	976.0	959.5		932.5
6.96	976.1	959.7	942.3	932.3
7.15	976.1	960.2		932.2
7.27	976.1	959.8		932.2
7.33	976.0	959.8		932.1
7.38	975.9	959.9		932.1
7.45	976.4	960.2		932.1
7.51	975.0	959.9		932.1
7.56	979.3	959.8		932.1
7.65	976.6	963.5		932.1
7.78	976.5	963.4		932.1
7.86	978.0	960.0		932.1
7.96				932.0
8.14				932.0
9.3				932.0
9.84				932.0
10.99				932.0
11.72				932.0
11.97				932.0

S3.3. Relative concentration from Raman spectra

The calculations for % relative concentration of POMs solution species was performed by deconvoluting the Raman bands observed in the Raman spectra (Section S3.1) using Voigt profiles and assigning characteristic Raman shifts as shown in Table 1 in the main paper.

The band area obtained from fitting Voigt profiles were used to calculate the %relative concentration for a given species at different pH values as follows:

$$\%\,relative\ concentration\ (PW_{12}) = \frac{Area(PW_{12})}{Total\ Area\ (W)} = \frac{Area(PW_{12})}{Area(PW_{12}) + Area(PW_{11}) + Area(WO_4)} \times 100 \quad (\mathrm{Eq.\,S1})$$

$$\%\,relative\ concentration\ (PW_{11}) = \frac{Area(PW_{11})}{Total\ Area\ (W)} = \frac{Area(PW_{11})}{Area(PW_{12}) + Area(PW_{11}) + Area(WO_4)} \times 100 \quad (Eq.S2)$$

$$\%\,relative\ concentration\ (WO_4) = \frac{Area(WO_4)}{Total\ Area\ (W)} = \frac{Area(WO_4)}{Area(PW_{12}) + Area(PW_{11}) + Area(WO_4)} \times 100 \quad (EqS3)$$

Similar calculations followed for pH speciation of SiW_{12} and H_2W_{12}.

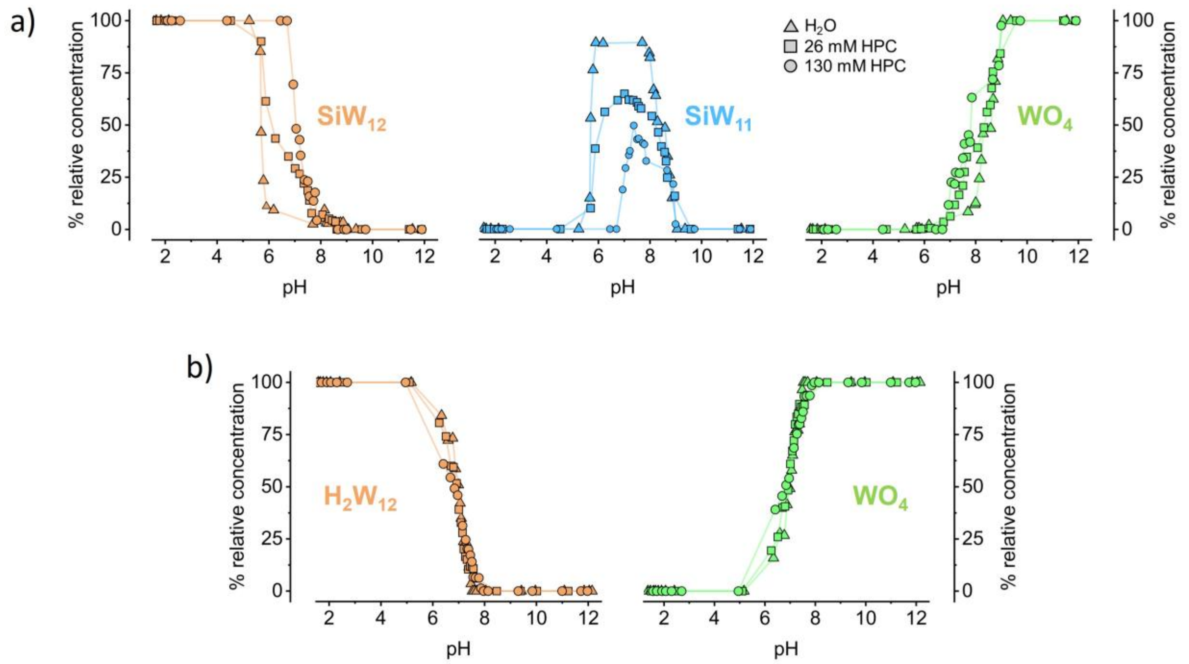

Figure S14. pH dependent speciation profiles of 5 mM Keggin POMs in water and with hydroxypropyl cellulose (HPC):]; a) shows stability range of SiW_{12} (left), SiW_{11} (center), and WO_4 (right) upon the hydrolysis of SiW_{12} b) shows stability range of H_2W_{12} (left) and WO_4 (right) upon the hydrolysis of H_2W_{12}. Data are shown for three different conditions: H_2O (▲), 26 mM HPC (■) and 130 mM HPC (●).

S3.4. Modelling Speciation of POMs

HySS2009 software[5] generates speciation diagrams based on chemical equilibria and formation constants. The program solves mass-balance equations iteratively using the Newton-Raphson method combined with Choleski factorization. For the calculations, the relevant reaction stoichiometry, mass-balance equations, and the experimental pH range were provided as input parameters in HySS2009. The formation constants (logβ) were then determined by iterative adjustment of logβ values until optimal agreement between simulated species distributions and Raman-derived concentration data was achieved. To estimate the uncertainty in the formation constants, the logβ values were iteratively adjusted to slightly overpredict or underpredict the experimental speciation. The average value and standard deviation of the formation constants (logβ) were obtained from four such iterative adjustments. The speciation diagrams shown in the Supporting Information (Figures S15–S17) were generated using the average logβ values obtained from these iterations.

In chemical equilibrium, the forward and reverse reactions take place at the same rate and the concentrations of reactants and products remain constant. Equation S4 shows a general reaction that is at chemical equilibrium.

$$aA + bB \rightleftharpoons cC + dD \qquad \text{(Eq. S4)}$$

The equilibrium constants K can be used to describe the relationship between products and reactants in a state of equilibrium.

$$K = \frac{[C]^c \cdot [D]^d}{[A]^a \cdot [B]^b} \qquad \text{(Eq. S5)}$$

To describe the speciation of POM species, we considered a minimal set of equilibria. The chemical equilibria involved in POM speciation are expressed in terms of formation constants.

For PW_{12} the following two equilibria are used*:

$$PO_4^{3-} + 12\,WO_4^{2-} + 24\,H^+ \rightleftharpoons 12\,H_2O + [PW_{12}O_{40}]^{3-} \qquad \text{(Eq. S6)}$$

$$PO_4^{3-} + 11\,WO_4^{2-} + 18\,H^+ \rightleftharpoons 9\,H_2O + [PW_{11}O_{39}]^{7-} \qquad \text{(Eq. S7)}$$

From these reaction equilibria, the mass balance equation was formulated and solved using the Newton-Raphson method to achieve a fit to the experimental data.[5]

Mass balance equations:

$$W{:}\,T_w = [WO_4^{2-}] + 12[PW_{12}] + 11[PW_{11}]$$

$$= [WO_4^{2-}] + 12\beta_1[PO_4^{3-}][PW_{12}][WO_4^{2-}]^{12}[H^+]^{24} + 11\beta_2[PO_4^{3-}][WO_4^{2-}]^{11}[H^+]^{18}$$

$$P : T_P = [PO_4^{3-}] + [PW_{11}] + [PW_{12}]$$

$$= [PO_4^{3-}] + \beta_2[PO_4^{3-}][WO_4^{2-}]^{11}[H^+]^{18} + \beta_1\,[PO_4^{3-}][WO_4^{2-}]^{12}[H^+]^{24}$$

Where formation constants for $[PW_{12}O_{40}]^{3-}$ is

$$\beta_1 = \frac{[PW_{12}]}{[PO_4^{3-}][WO_4^{2-}]^{12}[H^+]^{24}} \qquad \text{(Eq. S8)}$$

Where formation constants for $[PW_{11}O_{39}]^{7-}$ is

$$\beta_2 = \frac{[PW_{11}]}{[PO_4^{3-}][WO_4^{2-}]^{11}[H^+]^{18}} \qquad \text{(Eq. S9)}$$

For SiW_{12} the following two equilibria are used:

$$SiO_4^{4-} + 12\ WO_4^{2-} + 24\ H^+ \rightleftharpoons 12\ H_2O + [SiW_{12}O_{40}]^{4-} \qquad \text{(Eq. S10)}$$

$$SiO_4^{4-} + 11\ WO_4^{2-} + 18\ H^+ \rightleftharpoons 9\ H_2O + [SiW_{11}O_{39}]^{8-} \qquad \text{(Eq. S11)}$$

Mass balance equations:

$$W: T_w = [WO_4^{2-}] + 12[SiW_{12}] + 11[SiW_{11}]$$

$$= [WO_4^{2-}] + 12\beta_1[SiO_4^{4-}][WO_4^{2-}]^{12}[H^+]^{24} + 11\beta_2[SiO_4^{4-}][WO_4^{2-}]^{11}[H^+]^{18}$$

$$Si : T_{Si} = [SiO_4^{4-}] + [SiW_{11}] + [SiW_{12}]$$

$$= [SiO_4^{4-}] + \beta_2[SiO_4^{4-}][WO_4^{2-}]^{11}[H^+]^{18} + \beta_1\ [SiO_4^{4-}][WO_4^{2-}]^{12}[H^+]^{24}$$

Where formation constants for $[SiW_{12}O_{40}]^{4-}$ is

$$\beta_1 = \frac{[SiW_{12}]}{[SiO_4^{4-}][WO_4^{2-}]^{12}[H^+]^{24}} \qquad \text{(Eq. S12)}$$

Where formation constants for $[SiW_{11}O_{39}]^{8-}$ is

$$\beta_2 = \frac{[SiW_{11}]}{[SiO_4^{4-}][WO_4^{2-}]^{11}[H^+]^{18}} \qquad \text{(Eq. S13)}$$

For H_2W_{12} the following equilibria was used*:

$$12\ WO_4^{2-} + 18\ H^+ \rightleftharpoons 8\ H_2O + [H_2W_{12}O_{40}]^{6-} \qquad \text{(Eq. S14)}$$

Mass balance equations:

$$W: T_w = [WO_4^{2-}] + 12[H_2W_{12}]$$

$$= [WO_4^{2-}] + 12\beta_1[WO_4^{2-}]^{12}[H^+]^{18}$$

Where formation constants for $[H_2W_{12}O_{40}]^{6-}$ is

$$\beta_1 = \frac{[H_2W_{12}]}{[WO_4^{2-}]^{12}[H^+]^{18}} \qquad \text{(Eq. S15)}$$

Additionally, the following isopolytungstate species equilibria were incorporated into the speciation modeling of Keggin POMs. Tungstate undergoes condensation reactions to form metatungstate ions as:

$$7\ WO_4^{2-} + 8\ H^+ \rightleftharpoons 4\ H_2O + [W_7O_{24}]^{6-} \qquad \text{(Eq. S16)}$$

$$10\ WO_4^{2-} + 16\ H^+ \rightleftharpoons 8\ H_2O + [W_{10}O_{32}]^{4-} \qquad \text{(Eq. S17)}$$

$$12\ WO_4^{2-} + 18\ H^+ \rightleftharpoons 8\ H_2O + [H_2W_{12}O_{40}]^{6-} \qquad \text{(Eq. S18)}$$

The formation constants (logβ) for Eq.13-15 are 68.5, 120.1 and 144.7 respectively and were obtained from following literature.[6,7]

The modeled concentration of the isopolytungstate species $[W_7O_{24}]^{6-}$, $[W_{10}O_{32}]^{4-}$, $[H_2W_{12}O_{40}]^{6-}$were added to the modeled fits of lacunary POMs $[SiW_{11}O_{39}]^{8-}$and $[PW_{11}O_{39}]^{7-}$ when fitting the experimental data for lacunary POMs

The total modelled concentrations were therefore expressed as:

$$[SiW_{11}O_{39}]^{8-}(total) = [SiW_{11}O_{39}]^{8-} + [W_7O_{24}]^{6-} + [W_{10}O_{32}]^{4-} + [H_2W_{12}O_{40}]^{6-}$$

$$[PW_{11}O_{39}]^{7-}(total) = [PW_{11}O_{39}]^{7-} + [W_7O_{24}]^{6-} + [W_{10}O_{32}]^{4-} + [H_2W_{12}O_{40}]^{6-}$$

*Formation equilibria reactions for PW_{12} and H_2W_{12} taken from references[6,7]

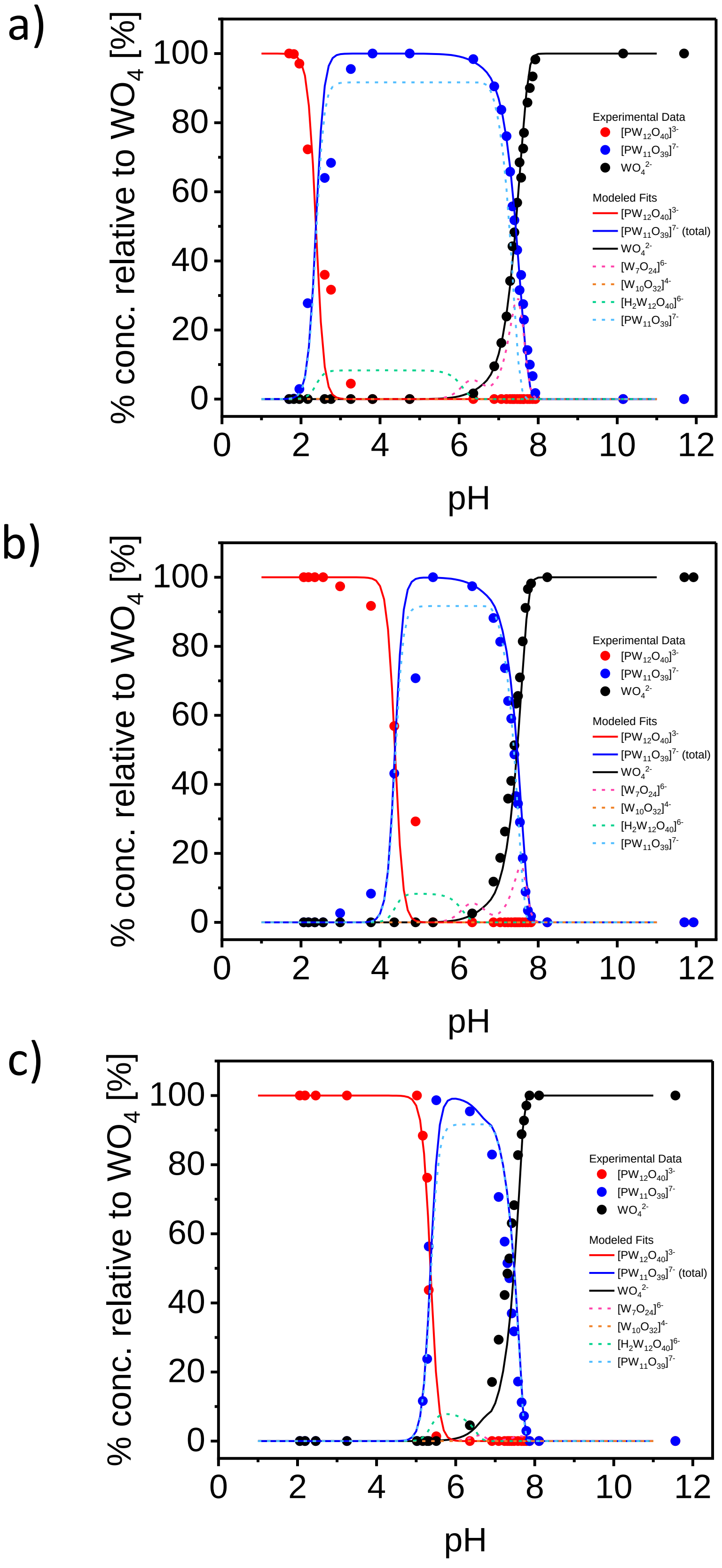

Figure S15. Tungstate species distribution for 5 mM PW_{12} in presence of a) H_2O, b) 26 mM HPC in H_2O, and c) 130 mM HPC using Raman spectra. The calculated curves represent the best fits to the experimental data based on the formation constants provided in Figure 2e.

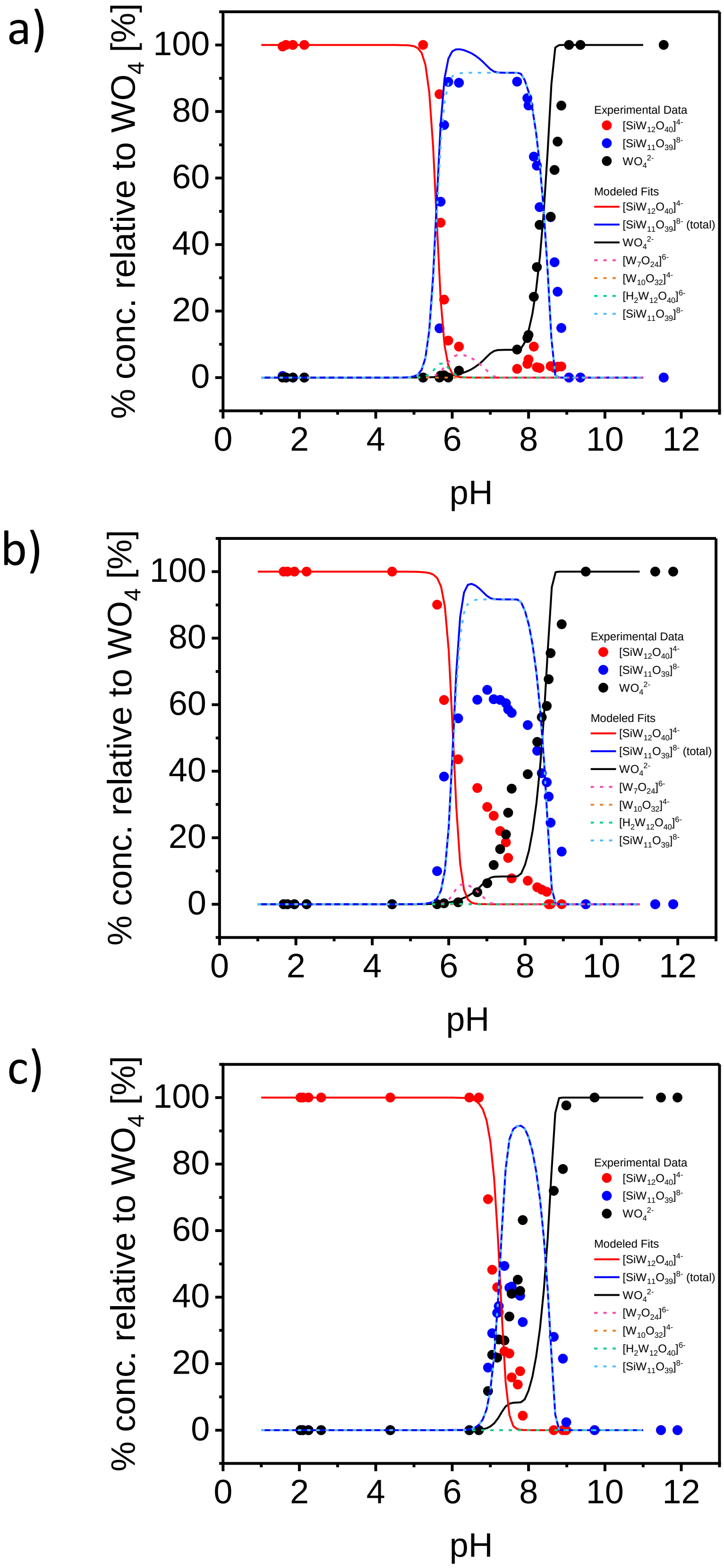

Figure S16. Tungstate species distribution for 5 mM SiW_{12} in presence of a) H_2O, b) 26 mM HPC, and c) 130 mM HPC, using Raman spectra. The calculated curves represent the best fits to the experimental data based on the formation constants provided in Figure 2e.

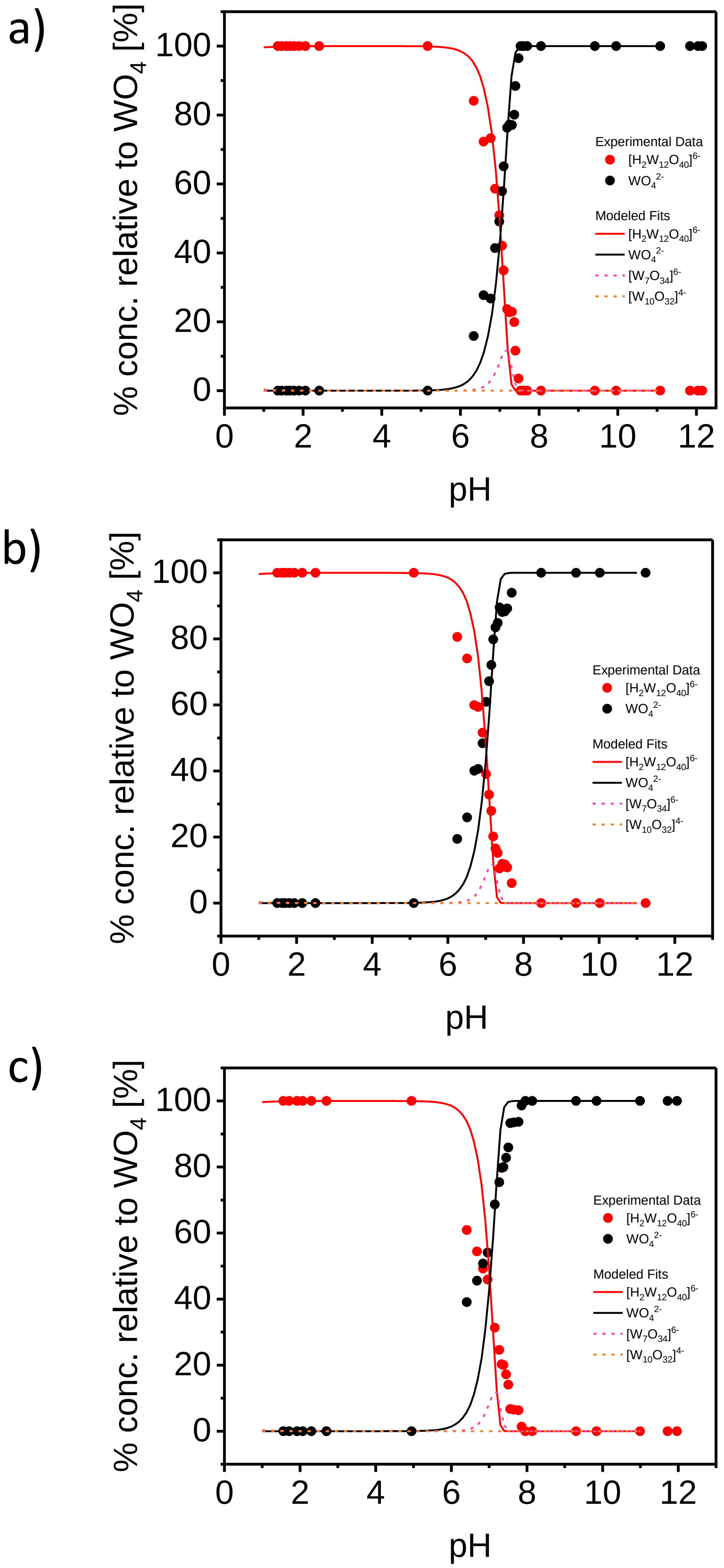

Figure S17. Tungstate species distribution for 5 mM H_2W_{12} in presence of a) H_2O, b) 26 mM HPC, and c) 130 mM HPC, using Raman spectra. The calculated curves represent the best fit to the experimental data based on the formation constants provided in Figure 2e.

S3.5. pH Speciation in H_2O and D_2O studied using Raman spectroscopy

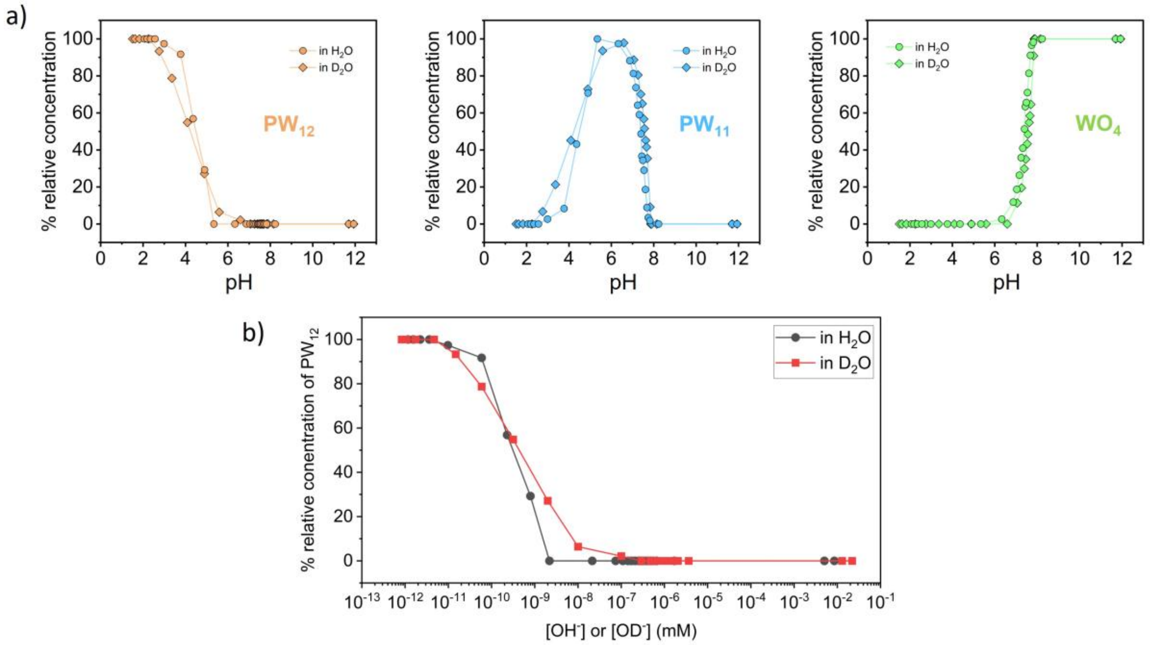

Figure S18. a) pH dependent profiles of 5 mM PW_{12} in presence of 26 mM HPC in H_2O and in D_2O. b) % relative concentration of PW_{12} as a function of OH^- and OD^- concentration.

During base-induced hydrolysis, the Keggin PW_{12} undergoes stepwise OH^- attack at bridging W–O–W bonds, resulting in hydrolytic removal of addenda W–O fragment and formation of lacunary PW_{11}, ultimately leading to complete decomposition into WO_4^{2-} and PO_4^{3-} ions. The pH dependent speciation profile of PW_{12} in the presence of 26 mM HPC (Figure S18a) shows a systematic shift of the $PW_{12}\leftrightarrow PW_{11}\leftrightarrow WO_4$ equilibria towards higher pH values in D_2O compared to H_2O, consistent with the apparent enhanced hydrolytic stability of the Keggin PW_{12} in the deuterated solvent. Importantly, when plotted as a function of hydroxide concentration, the complete decomposition of PW_{12} requires higher $[OD^-]$ than $[OH^-]$ (See Figure S18b).

Because the hydrolytic decomposition of PW_{12} includes the breakage of OH (OD) bonds we compare the respective bond dissociation energies (BDE). The O–D bond (BDE = 5.19 ± 0.01 eV, 500.76 kJ/mol) is more stable as reflected by a higher bond dissociation energy than the O–H bond (BDE = 5.28 ± 0.01 eV, 509.44 kJ/mol).[8] Reaction of O–D with PW_{12} is therefore thermodynamically less favored than the same reaction with O–H, which explains this stabilization effect.

S3.6. Speciation in two phases

The modelling of pH speciation of SiW_{12} in presence of HPC does not provide a proper fit. However, SiW_{12} in the presence of HPC exists as free SiW_{12} in water and bound SiW_{12} to HPC. Hence, the speciation of SiW_{12} in the presence of HPC occurs in two different phases: the HPC phase and the water phase.

So, we performed modeled fits by considering two independent speciation profiles in water phase (free SiW_{12}) and in the HPC phase (bound SiW_{12}) and added them to give the best fit to the observed speciation.

To this end, we first modeled the speciation of free SiW_{12} using the formation constants obtained for SiW_{12} in water. and then modelled the speciation for bound SiW_{12} to obtain the best fit. The ratio of free SiW_{12}: bound SiW_{12} was set to 3:2.

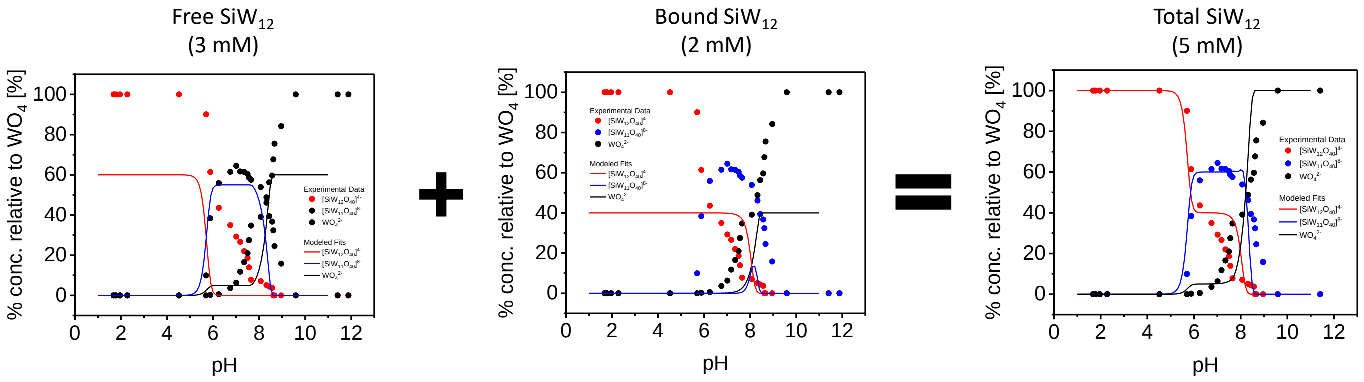

Figure S19. Tungstate species distribution for SiW_{12} in presence of 26 mM HPC (from left to right): 3 mM SiW_{12} in water phase, 2mM SiW_{12} in HPC phase and 5 mM SiW by addition of water and bound phase.

Formation constant values obtained are as follows:

POM species	log β	
	In water phase (free SiW_{12})	In HPC phase (bound SiW_{12})
SiW_{12}	205	219
SiW_{11}	168	168

S4. ^{1}H NMR Measurements

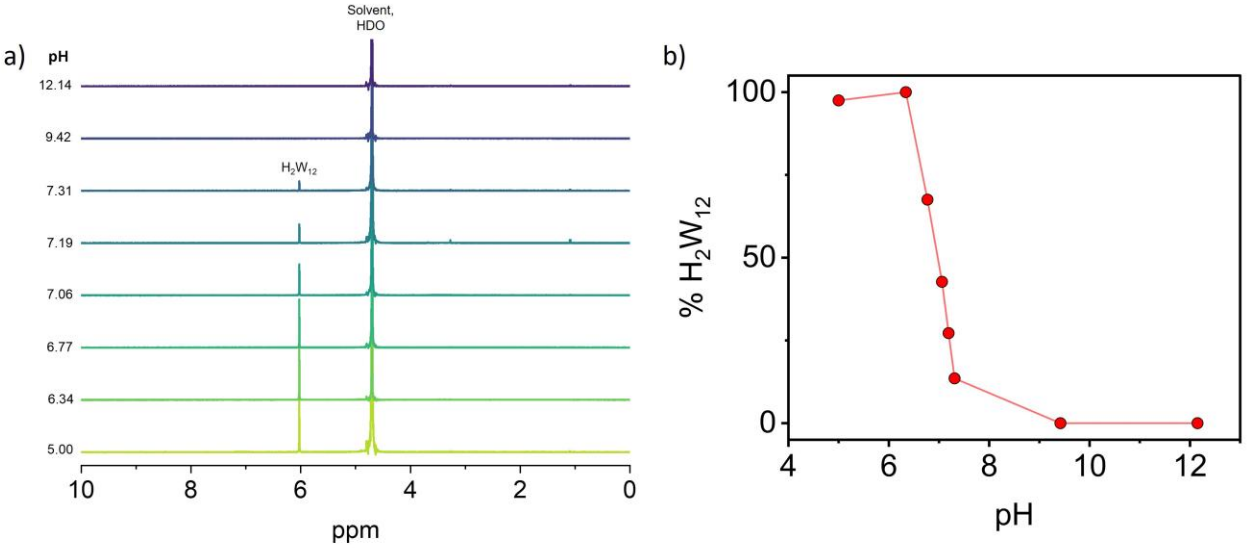

Figure S20. a) ^{1}H NMR spectra of 5 mM H_2W_{12} in D_2O at different pH levels, adjusted with NaOD. b) %H_2W_{12} distribution in 5 mM H_2W_{12} in D_2O at different pH levels from ^{1}H NMR spectra.

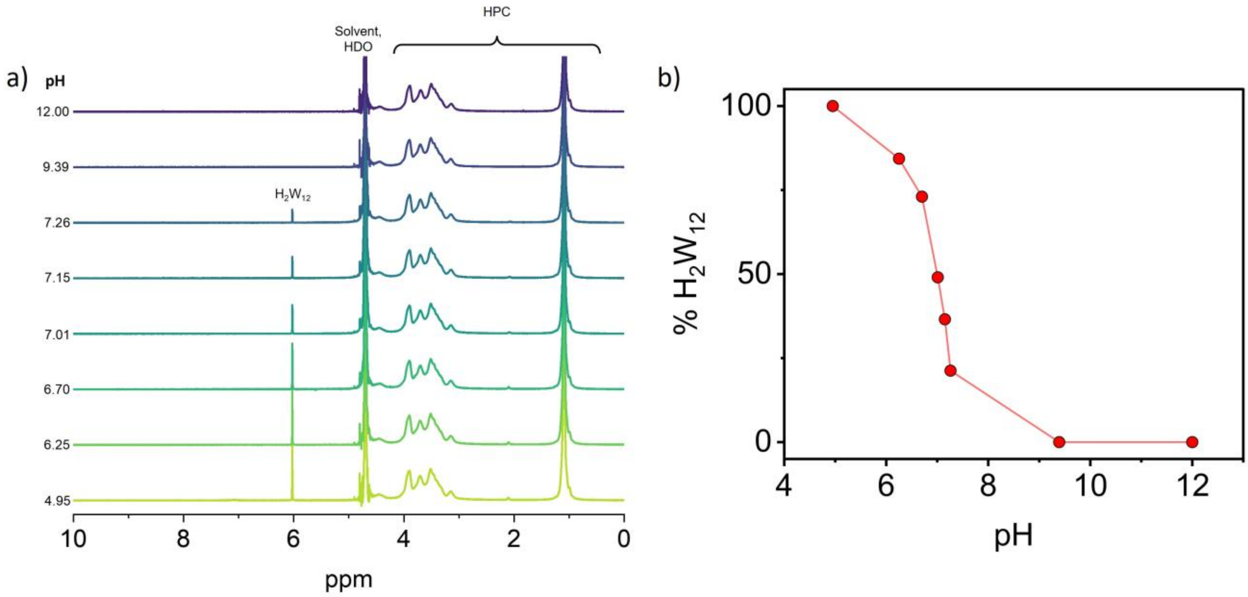

Figure S21. a) ^{1}H NMR spectra of 5 mM H_2W_{12} in presence of 26 mM HPC in D_2O at different pH levels, adjusted with NaOD. b) %H_2W_{12} distribution in 5 mM H_2W_{12} in presence of 26 mM HPC in D_2O at different pH levels from ^{1}H NMR spectra.

S5. ^{31}P NMR Measurements

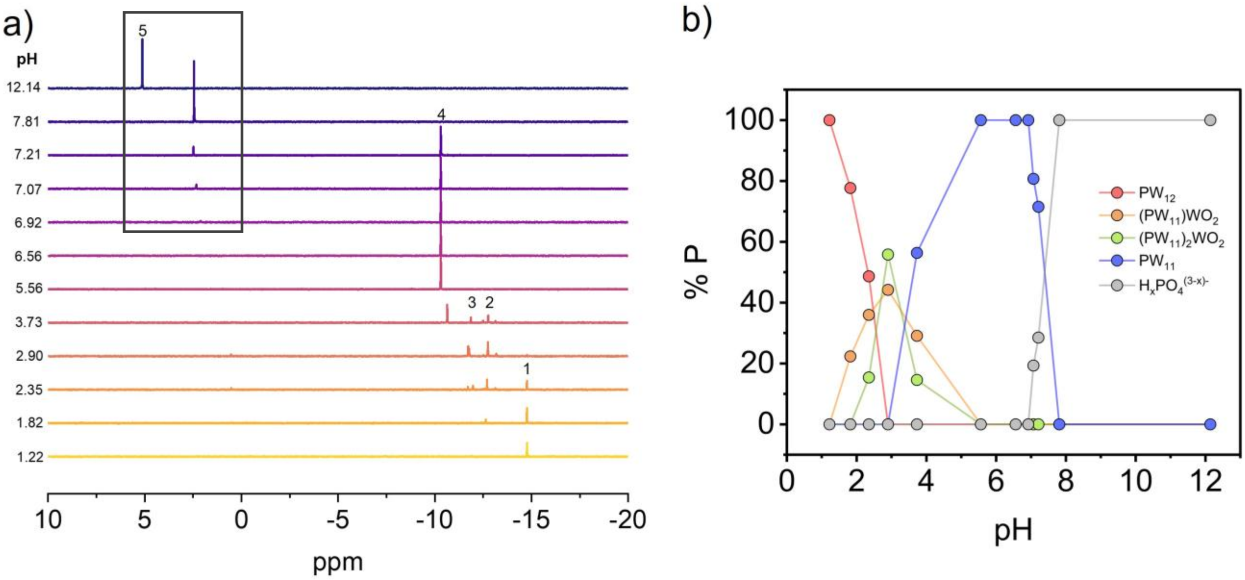

Figure S22. a) Solution ^{31}P NMR spectra of 5 mM PW_{12} in D_2O at different pH levels, adjusted with NaOD. b) Phosphate species distribution in 5 mM PW_{12} in D_2O at different pH levels from ^{31}P NMR spectra.

Table S10. Assignment of ^{31}P NMR signals from hydrolysis of 5 mM PW_{12} in D_2O.

Label	Species	Anion	^{31}P shift [ppm]
1	PW_{12}	$[PW_{12}O_{40}]^{3-}$	-14.7
2	$(PW_{11})WO_2$	$[(PW_{11}O_{39})WO_2]^{5-}$	-12.6 to -12.7
3	$(PW_{11})_2WO_2$	$[(PW_{11}O_{39})_2WO_2]^{12-}$	-12.0 to -11.7
4	PW_{11}	$[PW_{11}O_{39}]^{7-}$	-10.6 to -10.3
5	$H_xPO_4^{(3-x)-}$	HPO_4^{2-}, PO_4^{3-}	2.3 to 5.1

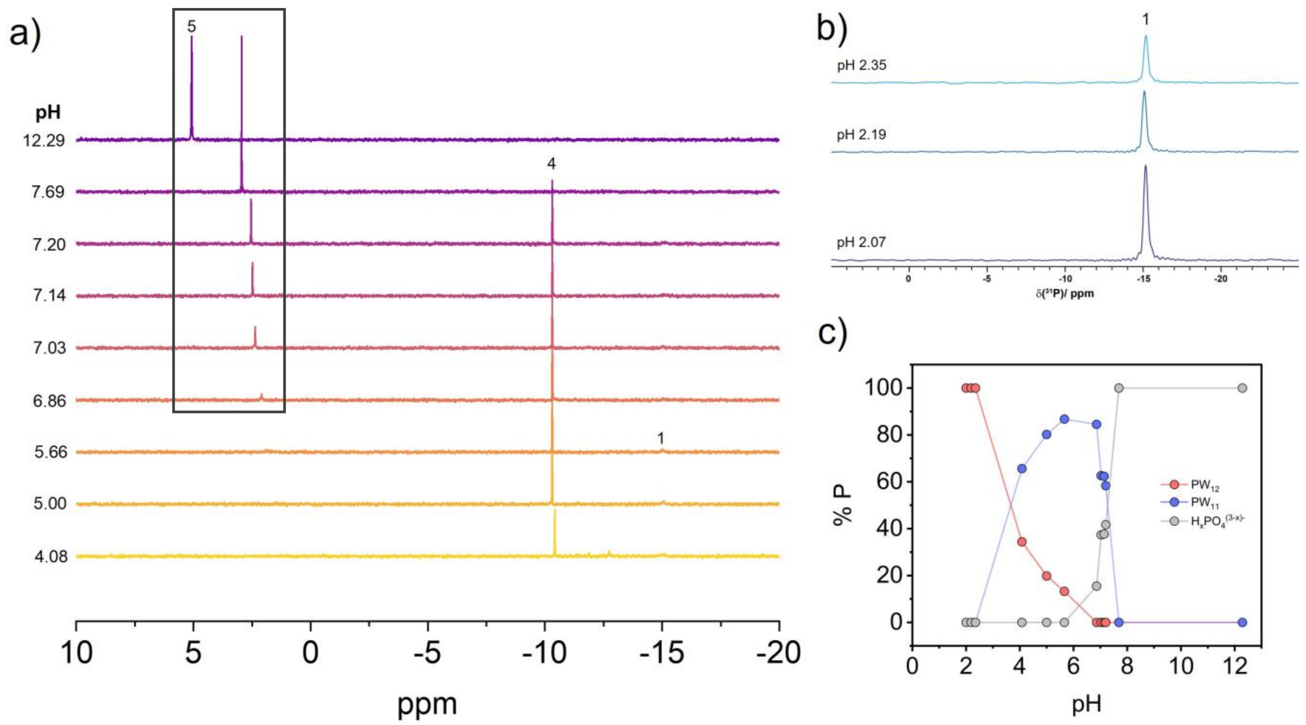

Figure S23. a) Solution ^{31}P NMR spectra of 5 mM PW_{12} in presence of 26 mM HPC in D_2O at different pH levels, adjusted with NaOD. b) Solid state ^{31}P NMR spectra of 5 mM PW_{12} in presence of 26 mM HPC in H_2O at different pH levels, adjusted with NaOH. c) Phosphate species distribution in 5 mM PW_{12} in presence D_2O/H_2O at different pH levels from ^{31}P NMR spectra.

Table S11. Assignment of ^{31}P NMR (solid state and solution) signals from hydrolysis of 5 mM PW_{12} in presence of 26 mM HPC.

Label	Species	Anion	^{31}P shift [ppm]
1	PW_{12}	$[PW_{12}O_{40}]^{3-}$	-15.2. to -14.7
4	PW_{11}	$[PW_{11}O_{39}]^{7-}$	-10.4 to -10.3
5	$H_xPO_4^{(3-x)-}$	HPO_4^{2-}, PO_4^{3-}	2.1 to 5.0

Peak positions determined from the following references.[7,9,10]

S6. Solvation free energies of POM species

We calculated the solvation free energies of different species during POM speciation using the approach described by Dullinger et al.[11] The molar solvation free energy (ΔG_{solv}) of an anion with charge z is expressed quantitatively as the sum of a favourable Born solvation energy (ΔG_{Born}) and an unfavourable surface energy term (ΔG_{surf}).

$$\Delta G_{solv} = \Delta G_{surf} + \Delta G_{Born} = 4\pi N_A {R_{ion}}^2 \gamma - \frac{N_A z^2 e^2}{8\pi\varepsilon_0}\left(1 - \frac{1}{\varepsilon_r}\right)\frac{1}{R_{ion}} \qquad (Eq. S19)$$

Where, γ is the surface tension of water (0.07197 N m^{-1}), ε_0 is the vacuum permittivity (8.854×10^{-12} C^2 kg^{-1} m^{-3} s^2), $\varepsilon_r$is the relative permittivity of water (80.2), z is the anion charge and R_{ion} is the van der Waals radius of the anion.

The van der Waals radii of the POM species were calculated from their van der Waals volume.[12]

Table S12. Calculated values for the van der Waals radii (R_{ion}) of the anions and their molar solvation free energies (ΔG_{solv}) at 25°C.

POM species	V_{ion} [nm^3]	R_{ion} [nm]	ΔG_{solv} [kJ mol^{-1}]
$[PW_{12}O_{40}]^{3-}$	0.62	0.529	-1014
$[SiW_{12}O_{40}]^{4-}$	0.63	0.532	-1908
$[H_2W_{12}O_{40}]^{6-}$	0.64	0.535	-4459
$[PW_{11}O_{39}]^{7-}$	0.6[1]	0.523	-6276
$[SiW_{11}O_{39}]^{8-}$	0.59[1]	0.52	-8293

[1] calculated using van der Waals radii of W and O

S7. Modeling of the SAXS and SANS profiles

The scattering intensity $I(q)$ for an isotropic sample containing identical, randomly oriented and non-interacting particles can be expressed by Eq. S20.

$$I(q) = \phi \cdot V \cdot \Delta\rho^2 \cdot P(q) + I_{bkg} \qquad \text{(Eq. S20)}$$

Here, ϕ is the volume fraction of the scattering object, V is the volume of one particle. The volume fraction can be rewritten as $\phi = n \cdot V$, where n is the number density of the scatterers. $\Delta\rho = |\rho_{scatterer} - \rho_{solvent}|$, is the scattering contrast which is defined as the difference in scattering length density (SLD) in SANS or electron density (ED) in SAXS between the scattering particle and the solvent, the form factor $P(q)$ describes the geometry of the particle and I_{bkg} is the q-independent scattering of the solvent (and incoherent scattering in SANS).

For samples containing interacting particles Eq.S20 can be written as

$$I(q) = \phi \cdot V \cdot \Delta\rho^2 \cdot P(q) \cdot S(q) + I_{bkg} \qquad \text{(Eq. S21)}$$

$S(q)$ is the structure factor which describes the particle arrangement that depends on particle-particle interactions.

For a sample containing different scattering particles, the scattering intensity is expressed as a sum over all objects i:

$$\textit{For non interacting species:} \quad I(q) = \sum_i \phi_i \cdot V_i \cdot \Delta\rho_i^2 \cdot P_i(q) + I_{bkg} \qquad \text{(Eq. S22)}$$

$$\textit{For interacting species:} \quad I(q) = \sum_i \phi_i \cdot V_i \cdot \Delta\rho_i^2 \cdot P_i(q) \cdot S_i(q) + I_{bkg} \qquad \text{(Eq. S23)}$$

All SANS-profiles were fitted with SASView 5.0.6 and SAXS profiles were fitted with SASView 5.0.6 and SASFit0.94.12. Both softwares use an equivalent expression for the scattered intensity. SASView uses the volume fraction of the scatterers ϕ, while SASFit uses the number density of scatterers n.

The ED_i (for SAXS) and SLD_i (for SANS) were calculated in order to determine the scattering contrast.

The electron densities EDs was calculated using Eq.S24.

$$ED_i = \frac{\sum_j a_j}{v_i} \cdot l_{Thomson} \qquad \text{(Eq. S24)}$$

Here, a_j is the number of electrons in the jth atom in the volume element v_i of the scatterer and $l_{Thomson}$ = 2.82 × 10^{-15} m is the Thomson length.

The scattering length densities SLDs was calculated using Eq.S25.

$$SLD_i = \frac{1}{v_i} \sum_j b_j \qquad \text{(Eq. S25)}$$

Here, b_j is the coherent scattering length of the jth atom.

The SLDs, EDs, a_j, b_j and $V_i$of all molecules used in this study are shown in Table S13.

Table S13. Molecular volumes, scattering length densities, scattering length, electron densities and number of electrons of all the compounds used in data fitting.

Compound	V_i [nm^3]	SANS		SAXS	
		b_j [fm]	SLD [10^{-6} Å^{-2}]	a_j	ED [10^{-6} Å^{-2}]
$[PW_{12}O_{40}]^{3-}$	0.62[1]	295.5	4.69	1223	54.7
$[PW_{11}O_{39}]^{7-}$	0.59[2]	284.9	4.75	1141	53.6
$[SiW_{12}O_{40}]^{4-}$	0.63[1]	294.5	4.78	1222	54.7
$[SiW_{11}O_{39}]^{8-}$	0.6[2]	283.9	4.43	1140	53.6
HPC	0.51[3]	41.1	0.81	208	11.6
H_2O	0.03[4]	-	-	10	9.4
D_2O	0.03[4]	19.1	6.33	-	-

[1] taken from reference[12] [2] calculated using van der Waals radii of W and O
[3] obtained from densimetry [4] calculated form pure solvent densities

S7.1. Fitting of SANS profiles

In SANS, the scattering contrast for both Keggin and Lacunary POM species matches with the solvent D_2O and the neutrons exclusively scatter due to the polymer HPC. As a result, the SANS profiles only contain information of HPC chains and HPC/POM aggregates. Eq.S20 qualitatively describes the two components HPC/D_2O and the three components HPC/POM/D_2O using known scattering contrast values and volume fractions and can be modelled using a cylindrical form factor.

$$I^{SANS}(q) = I_{HPC-POM} + I_{bkg}$$

$$I^{SANS}(q) = \phi_{HPC-POM} \cdot V_{HPC-POM} \cdot {\Delta\rho_{HPC-POM}^{SANS}}^2 \cdot P_{cylinder}(q, r, L) + I_{bkg} \qquad \text{(Eq. S26)}$$

For the HPC-POM aggregates, the volume fraction $\phi_{HPC-POM}$ was fixed to the known volume fraction of HPC ϕ_{HPC}. The contrast factor $\Delta\rho_{HPC-POM}^{SANS}$ and the radius of the cylinder were kept as free parameters. Meanwhile, the cylinder length was set to an arbitrarily large value of 100 nm, beyond the modelled q-region.

Table S14. SANS-Fit parameters used for fitting Eq.S26 (SASView)

**Initial sample composition**			**pH**		**SANS-fit parameters**				
					**Cylinder form factor**				
c(HPC) [mM]	POM	c(POM) [mM]		I_{bkg} [cm^{-1}]	$\phi_{HPC-POM}$	SLD_{D2O} 10^{-6} [Å^{-2}]	$SLD_{HPC-POM}$ 10^{-6} [Å^{-2}]	r [nm]	L [nm]
26	-	0	7	0	0.0088	6.3	0.81	0.46	100
26	PW_{12}	5	2.5	0.012	0.0088	6.3	2.4	1.29	100
26	PW_{12}	5	3.5	0.008	0.0088	6.3	2.4	1.27	100
26	PW_{12}	5	4.5	0.007	0.0088	6.3	2.4	1.11	100
26	PW_{12}	5	7	0	0.0088	6.3	2	0.74	100
26	PW_{12}	5	12	0	0.0088	6.3	0.81	0.46	100
26	SiW_{12}	5	1.5	0	0.0088	6.3	2.91	1.3	100
26	SiW_{12}	5	9	0	0.0088	6.3	1.01	0.48	100
26	SiW_{12}	5	12	0	0.0088	6.3	0.83	0.46	100

S7.2. Fitting of SAXS profiles for POM species in water

The scattering intensity $I(q)$ of individual POM species in water was measured using SAXS. The form factor of the POM species was computed in Crysol within ATSAS software using crystal structure data of the POMs.[13] Crysol computes the form factor by atomic distances in the POM structure according to the Debye equation (Eq.S27).

$$I(q) = \sum_i \sum_j b_i\, b_j \frac{\sin(q r_{ij})}{q r_{ij}} \qquad \text{(Eq. S27)}$$

Here, r_{ij} is the distance between scatterers (atoms) i and j in nm and b is the scattering length for the atom.

The scattering data fitted was obtained after subtraction of empty capillary background and solvent H_2O signal.

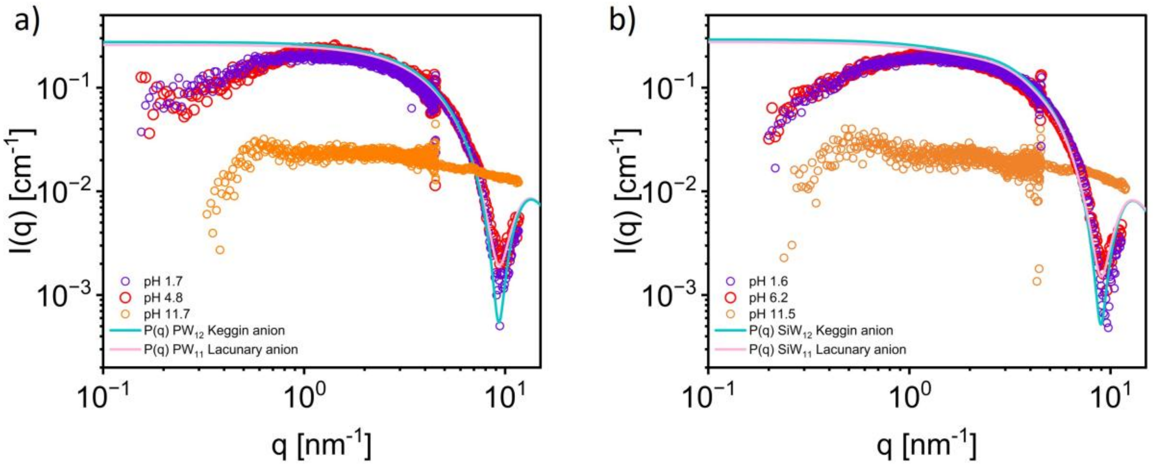

Figure S24. SAXS profiles for a) 5 mM PW_{12} in H_2O at three different pH values exclusively representing solution species Keggin PW_{12} (pH 1.7), lacunary PW_{11} (pH 4.8) and tungstate ion WO_4 (pH 11.7). b) 5 mM SiW_{12} in H_2O at three different pH values exclusively representing solution species Keggin SiW_{12} (pH 1.6), lacunary SiW_{11} (pH 6.2) and tungstate ion WO_4 (11.5). Solid line represents form factor *P(q)* fits for the Keggin and lacunary POM that can be distinguished based on the depth of the first minimum at 9 nm^{-1}.

S7.3. Fitting of SAXS profiles

Since the plenary Keggin and lacunary Keggin POM have a high electron density contrast with H_2O, they produce significant scattering in SAXS. During pH dependent speciation, accurately modeling the SAXS profiles requires accounting for all scattering contributions from the species present at a given pH. These scattering contributions includes: HPC/POM assemblies, individual POMs (lacunary and plenary) and interactions among them in solution. The scattering intensity for this system at any pH can be written as individual contributions from different species in the solution.

$$I^{SAXS}(q) = I_{P\,POM} + I_{L\,POM} + I_{HPC-POM} + I_{bkg} \quad \text{(Eq. S28)}$$

Here, $I_{P\,POM}$ is the scattered intensity of plenary Keggin POMs, $I_{L\,POM}$ the scattered intensity due to lacunary Keggin POM, $I_{HPC-POM}$ is the scattered intensity due to HPC/KegginPOM aggregates and I_{bkg} a background intensity.

We modeled the SAXS profiles in different pH regimes as follows:

1.) At low pH, when plenary Keggin POM is the only POM species present in the solution with HPC, the total scattered intensity can be written as:

$$I^{SAXS}(q) = I_{P\,POM} + I_{HPC-POM} + I_{bkg}$$

Using Eq.S22, the intensity $I^{SAXS}(q)$ is then expressed as

$$I^{SAXS}(q) = \phi_{P\,POM} \cdot V_{P\,POM} \cdot \Delta\rho_{P\,POM}^{SAXS\ 2} \cdot P_{sphere}(q,r) +$$
$$\phi_{HPC-POM} \cdot V_{HPC-POM} \cdot \Delta\rho_{HPC-POM}^{SAXS\ 2} \cdot P_{cylinder}(q,r,L) + I_{bkg} \quad \text{(Eq. S29)}$$

For plenary Keggin POM:
- The volume fraction $\phi_{P\,POM}$ was calculated from its initial concentration.
- A spherical form factor $P_{sphere}(q,r)$ was used to model the shape of plenary Keggin POMs, with radius r taken from literature and hence a fixed parameter.

For HPC/POMs aggregates:
- The volume fraction $\phi_{HPC-POM}$ was fixed to the known volume fraction of HPC ϕ_{HPC}.
- A cylinder form factor $P_{cylinder}(q,r,L)$ with radius r and length L was used to model their shape.
- The radius r and the scattering contrast $\Delta\rho_{HPC-POM}^{SAXS}$ were treated as fitting parameters.

2.) At low pH, when the plenary POM was partially hydrolyzed into lacunary POM. The scattered intensity can be written exactly like Eq.S28

$$I^{SAXS}(q) = I_{P\,POM} + I_{L\,POM} + I_{HPC-POM} + I_{bkg}$$

However, as shown in Section S7.2, plenary and lacunary POMs exhibit similar scattering profiles and have an almost indistinguishable form factor $P(q)$ in the mid- and low-q regions. Therefore, $I_{P\,POM} + I_{L\,POM} = I_{POMs}$ is valid and results in Eq. S29.
In this case, the effective volume fraction ϕ_{POMs} includes contributions from both the plenary POM and the lacunary POM, and is assumed to be equal to the initial volume fraction of the plenary Keggin POM (ie. before hydrolysis).

3.) At intermediate pH, when the plenary POM has completely hydrolyzed to lacunary POM or when lacunary POM has partially hydrolyzed to tungstate and phosphate/silicate ions. The tungstate and phosphate/silicate ions only contribute to the background scattering. Hence, the scattered intensity can be written as

$$I^{SAXS}(q) = I_{L\,POM} + I_{HPC} + I_{bkg}$$

Importantly, the scattered intensity of HPC arises exclusively from the HPC-chains (with known contrast and volume fraction) as there is no interaction with the lacunary POM species.

We used Eq.S21 to express $I^{SAXS}(q)$ as

$$I^{SAXS}(q) = n_{L\,POM} \cdot V_{L\,POM}^{\ 2} \cdot \Delta\rho_{L\,POM}^{SAXS\ 2} \cdot P_{sphere}(q,r) \cdot S_{HMSA}(q, r_{HS}, \phi, Z, I\,) +$$

$$n_{HPC} \cdot {V_{HPC}}^2 \cdot {\Delta\rho_{HPC}^{SAXS}}^2 \cdot P_{cylinder}(q, r, L) + I_{bkg} \quad \text{(Eq.S30)}$$

We used SASFit0.94.12 to model SAXS profiles using Eq.S30. The equation uses number densities $n_{L\,POM}$ and $n_{HPC-POM}$ instead of volume fraction ϕ.

For lacunary POM:

- The volume fraction $\phi_{L\,POM}$ (and number density $n_{L\,POM}$) can be determined by calculating the concentration of lacunary POM based on its percentage abundance from the speciation diagram.
- A spherical form factor $P_{sphere}(q, r)$ was used to model the shape of lacunary POM with radius r similar to that of Keggin POM.
- The Hayter Mean Sphere Approximation (HMSA) structure factor $S_{HMSA}(q, r_{HS}, \phi, Z, I)$ models the electrostatic repulsion between lacunary POMs at low q and depends on its hard sphere radius r_{HS}, volume fraction ϕ, charge on lacunary POM Z, and concentration of screening salt I.

For HPC/POMs aggregates the modeling is same as mentioned in 1), with volume density $n_{HPC-POM}$ as a free parameter.

4.) At high pH, when lacunary POM has completely hydrolyzed into smaller anions, the scattered intensity can be written as

$$I^{SAXS}(q) = I_{HPC} + I_{bkg}$$

$$I^{SAXS}(q) = \phi_{HPC} \cdot V_{HPC} \cdot {\Delta\rho_{HPC}^{SAXS}}^2 \cdot P_{cylinder}(q, r, L) + I_{bkg} \quad \text{(Eq.S31)}$$

The smaller anions such as WO_4^{2-}, PO_4^{3-} or SiO_4^{4-} only contribute to the background scattering.[14] Hence, the scattered intensity at this pH can simply be modeled using a cylindrical form factor $P_{cylinder}(q, r, L)$.

Model fits by both SAXS and SANS agree closely on the dimensions of the HPC/POM aggregates. SANS exclusively probes HPC, while SAXS probes HPC as well as the different POM species in solution and their interactions. However, SAXS corresponds more closely to the Raman data as the solvent is H_2O. In SANS, D_2O could have additional stabilization effects on the plenary Keggin POM (See Section S3.5).

All the fit parameters are compiled below:

Table S15. SAXS-Fit parameters used for fitting Eq.S29 (Method 1 and 2) (SASView)

Initial sample composition			pH	I_{bkg} [cm^{-1}]	SAXS fit parameters at low pH using fitting method 1 and 2								
					Sphere fit				Cylinder fit				
c(HPC) [mM]	POM	c(POM) [mM]			$\phi_{K\,POM}$	ED_{H2O} 10^{-6} [Å^{-2}]	$ED_{K\,POM}$ 10^{-6} [Å^{-2}]	$r_{K\,POM}$ [nm]	$\phi_{HPC-POM}$	ED_{H2O} 10^{-6} [Å^{-2}]	$ED_{HPC-POM}$ 10^{-6} [Å^{-2}]	r [nm]	L [nm]
26	PW_{12}	5	2.5	0.020	0.00187	9.4	54.7	0.44	0.0088	9.4	16.5	1.25	100
26	PW_{12}	5	3.5	0.021	0.00187	9.4	54.7	0.45	0.0088	9.4	16.5	1.19	100
26	PW_{12}	5	4.5	0.020	0.00187	9.4	54.7	0.46	0.0088	9.4	15.8	0.94	100
26	SiW_{12}	5	1.7	0.024	0.00189	9.4	54.7	0.49	0.0088	9.4	14.9	1.12	100

Table S16. SAXS-Fit parameters used for fitting Eq.S30 (Method 3) (SASFit))

Initial sample composition			pH	SAXS fit parameters at intermediate pH using fitting method 3													
				Sphere fit				HMSA structure factor						Cylinder fit			
c(HPC) [mM]	POM	c(POM) [mM]		I_{bkg} [cm^{-1}]	$n_{L\,POM}$ ($\phi_{L\,POM}$) [nm^{-3}]	$ED_{L\,POM} - ED_{H2O}$ 10^{-6} [Å^{-2}]	$r_{L\,POM}$ [nm]	r_{HS} [nm]	Z	ϕ	T [K]	I [mM]	ε	$n_{HPC-POM}$ [nm^{-3}]	$ED_{HPC-POM} - ED_{H2O}$ 10^{-6} [Å^{-2}]	r [nm]	L [nm]
26	PW_{12}	5	5.4	0.023	0.003 (0.00187)	44.2	0.53	0.6	7	0.0018	298	3	81	0.00016	5.83	0.46	30
26	PW_{12}	5	7.0	0.024	0.002 (0.0012)	44.2	0.52	0.54	7	0.0012	298	3	81	0.00016	5.3	0.46	30
26	SiW_{12}	5	7.6	0.026	0.0018 (0.0011)	44.2	0.52	0.53	7	0.0012	298	3	81	0.0002	5.2	0.47	30

Table S17. SAXS-Fit parameters used for fitting Eq.S31 (Method 4) (SASview)

Initial sample composition			pH		SAXS fit parameters at high pH using fitting method 4				
					Cylinder fit				
c(HPC) [mM]	POM	c(POM) [mM]		I_{bkg} [cm^{-1}]	$\phi_{HPC-POM}$	ED_{H2O} 10^{-6} [Å^{-2}]	$ED_{HPC-POM}$ 10^{-6} [Å^{-2}]	r [nm]	L [nm]
26	-	-	7.	0.020	0.0088	9.4	11.6	0.46	100
26	PW_{12}	5	12	0.032	0.0088	9.4	11.8	0.47	100
26	SiW_{12}	5	12	0.033	0.0088	9.4	11.8	0.53	100

S7.4. SAXS profile for HPC/PW_{12}

We performed complementary SAXS measurements to render the SANS results more robust. The SAXS data was in close agreement with SANS but probed both HPC and POM species. In SAXS, the POM species scatter more strongly than HPC due to higher electron density contrast with H_2O. The SAXS profile of 26 mM HPC was well described by a cylindrical form factor with a radius of 0.46 nm, in agreement with the value obtained from SANS. The addition of PW_{12} at pH 2.5 produced a shoulder at $q^* = 0.3$ nm^{-1} and a q^{-1} dependence at high q, closely matching SANS results and confirming consistent detection of PW_{12}/HPC aggregates. The scattering profile for PW_{12}/HPC aggregates was best described using a model fit combining spherical and cylindrical form factors, yielding a cylinder radius of 1.25 nm for the aggregates. The scattering intensity decreased with increasing pH from 2.5 to 4.5, confirming the pH-induced decrosslinking of PW_{12}/HPC aggregate. At pH 5.4, when a complete conversion to PW_{11} is achieved, the scattering profile can be modeled using a sum of cylinder form factor and a sphere form factor with a Hayter mean sphere approximation (HMSA) structure factor accounting for repulsion between lacunary PW_{11}. The model fit yielded a cylinder radius of 0.46 nm, matching the radius of free HPC chains in solution. The scattering profile at this pH can also be described as a sum of the individual intensities of 26 mM HPC and 5 mM PW_{11} (Figure S27), indicating that both species are uncorrelated and non-interacting in the solution. A similar scattering behavior occurred at pH 7, where PW_{11} is partially hydrolyzed. At pH 12, where PW_{11} has completely hydrolyzed to tungstate and phosphate ions, the scattered intensity can simply be modeled using a cylindrical form factor with a radius of 0.46 nm. The slight increase in the scattered intensity was attributed to background contributions from the tungstate and phosphate ions. The fitting procedure used at different pH values can be found in Section S7.3.

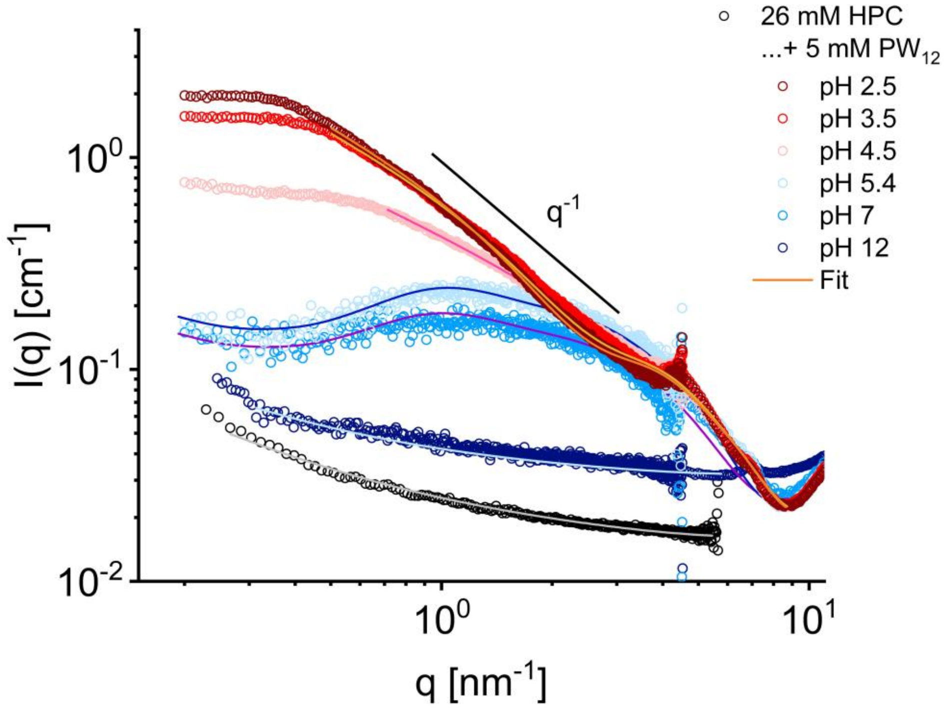

Figure S25. SAXS profiles at various pH for 26 mM HPC with 5 mM PW_{12} in H_2O. Solid lines represent fits for the data.

Additionally, for PW_{12}, we qualitatively observed that the presence of HPC may weakly promote PW_{12}-PW_{12} aggregation. First, the SAXS profile of 5 mM PW_{12} in water (See Figure S24) clearly shows a broad maximum at around $q_{PW12} \approx 1$ nm^{-1} which corresponds to a mean PW_{12}-PW_{12} distance of $d_{PW12} \approx 6.3$ nm. If we consider PW_{12} in a cubic lattice with d ≈ 6.3 nm, this spacing corresponds to a PW_{12} concentration of ≈ 6.7 mM in water (close to the experimental concentration of 5 mM, deviation may arise due to uncertainty in determination of exact peak position). This analysis confirmed that PW_{12} does not aggregate or cluster in water.

Upon addition of 26 mM HPC to 5 mM PW_{12} at pH 2.5 (See Figure S25), a weak correlation peak/shoulder appears at higher q value of q_{PW12} = 4.2 nm^{-1}, which is absent in case of SiW_{12} (Figure S26b). This q_{PW12} peak corresponds to a mean PW_{12}-PW_{12} distance of 1.5 nm, within the HPC/PW_{12} aggregates. Considering a PW_{12} radius of r_{PW12} = 0.45 nm, the gap between neighboring POMs is approximately 0.6 nm, which may be partially occupied by HPC segments. Notably, this correlation peak q_{PW12} disappears on conversion of Keggin PW_{12} to lacunary PW_{11}. Such PW_{12}-PW_{12} could potentially contribute to the stronger viscosifying effect, cloud point elevation and improved hydrolytic stability of Keggin PW_{12} in presence of HPC, since these aggregates could behave as stronger superchaotropic clusters with even lower charge density. Nevertheless, from the SAXS data, this contribution appears to be minor compared with the dominant superchaotropic interaction of individual PW_{12} anions with HPC, particularly at low concentrations (5 mM).

S7.5. SANS and SAXS profile for HPC/SiW_{12}

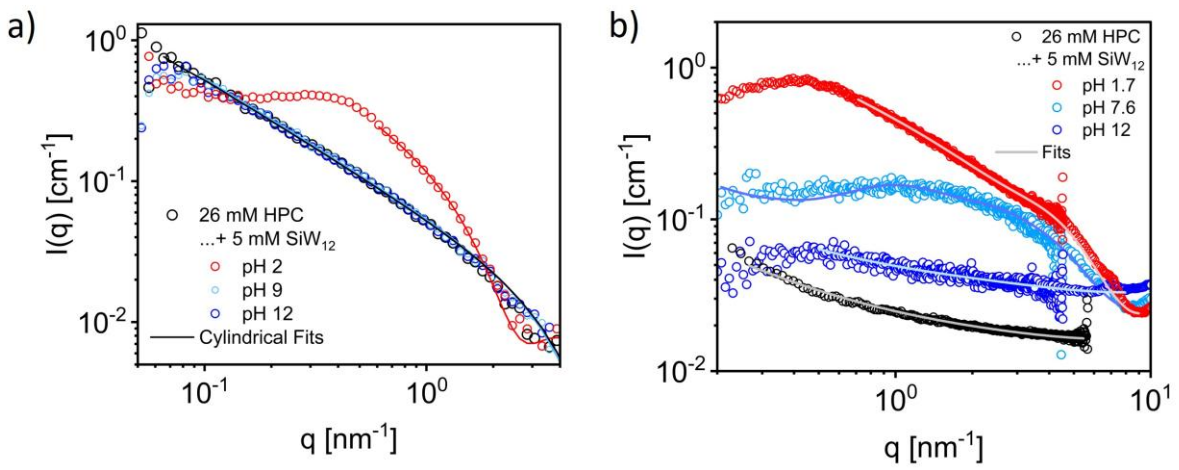

Figure S26. d) SANS and e) SAXS profiles at various pH for 26 mM HPC with 5 mM SiW_{12} in D_2O and H_2O respectively. Solid lines represent fits for the data.

S7.6. PW_{11} as non-interacting species in solution

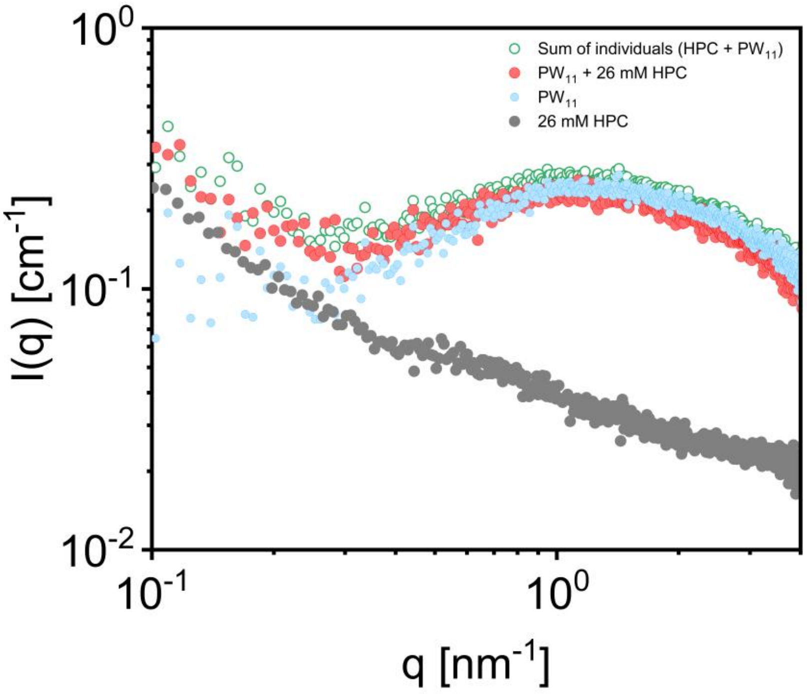

Figure S27. d) SAXS profile of HPC, PW_{11} in water and PW_{11} with HPC showing no interaction of the lacunary PW_{11} with HPC as the intensity for PW_{11} with HPC can be well described by the sum of individual intensities of PW_{11} and HPC. SAXS measurement performed on 5 mM PW_{12} / 26 mM HPC solution at pH 5.4, 5 mM PW_{12} solution at pH 5 and 26 mM HPC solution at pH 7.

S8. Coupled equilibria

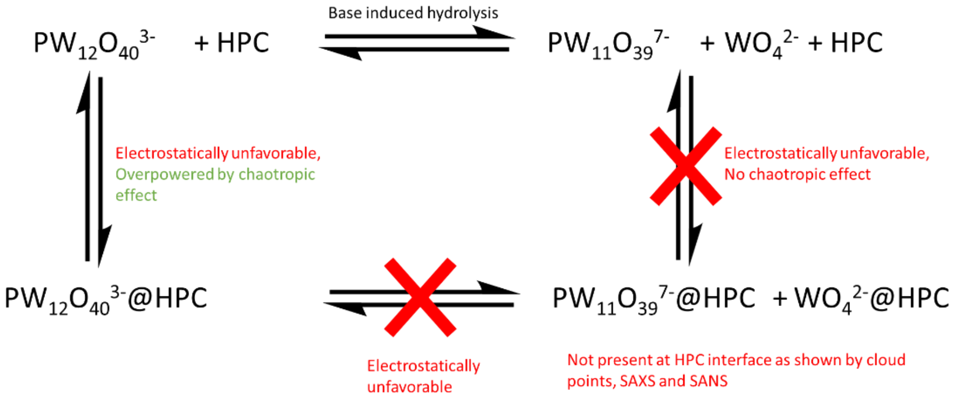

Scheme S1.Equilibria involved between $PW_{12}O_{40}^{3-}$, $PW_{11}O_{39}^{7-}$, WO_4^{2-} and HPC. The equilibria with red crosses are negligible in this system. Electrostatically unfavorable refers to calculations based on the Born energy, see eq.S19, either increasing the charge from plenary to lacunary POM or by decreasing the dielectric constant from water to HPC.

Scheme S1 shows the coupled equilibria of POM-HPC binding and POM hydrolysis. The chaotropic binding of PW_{12} to HPC along with direct POM-HPC interactions (H-bonding, ion-dipole interactions) lead to a stabilization effect against base induced hydrolysis. While the scheme is made on the example of PW_{12} it applies equally to SiW_{12}. For H_2W_{12}, the POM-HPC binding equilibrium is negligible and would be crossed out. Accordingly, no stabilization occurs in agreement with our data.

S9. Rheology

Zero shear viscosities (η_0) and terminal relaxation time ($\tau_{terminal}$) was extracted by fitting the flow curves with Carreau-Yasuda model according to Eq.S32, where $\dot{\gamma}$ is the shear rate and p is the power law exponent.

$$\eta(\dot{\gamma}) = \frac{\eta_0}{(1+(\tau_{terminal}\cdot\dot{\gamma})^2)^p} \qquad \text{(Eq. S32)}$$

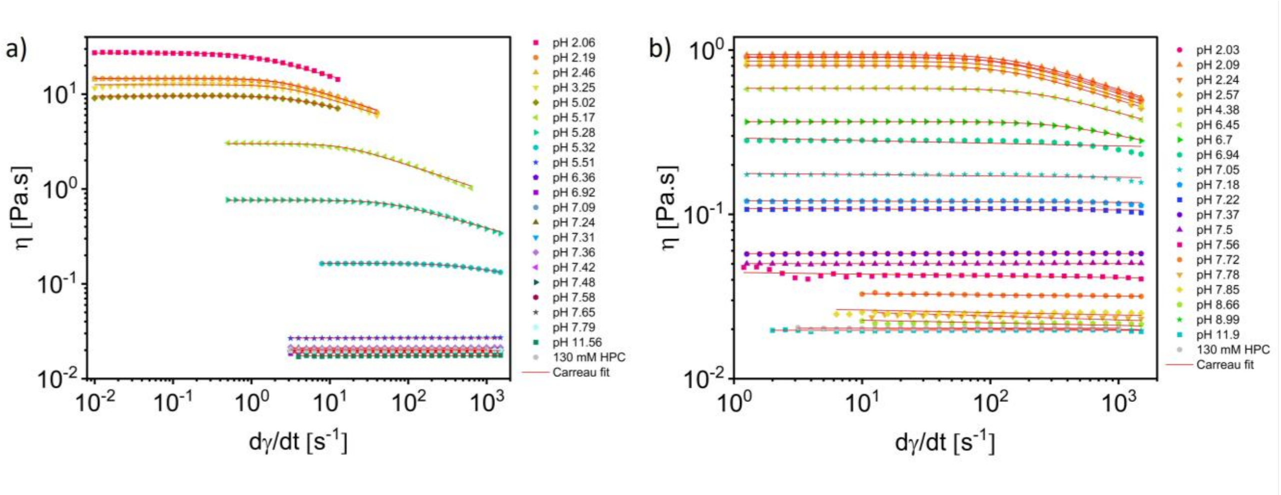

Figure S28. Flow curves of a) 5 mM PW_{12} and b) 5 mM SiW_{12} in presence of 130 mM HPC in H_2O at different pH values, adjusted using NaOH.

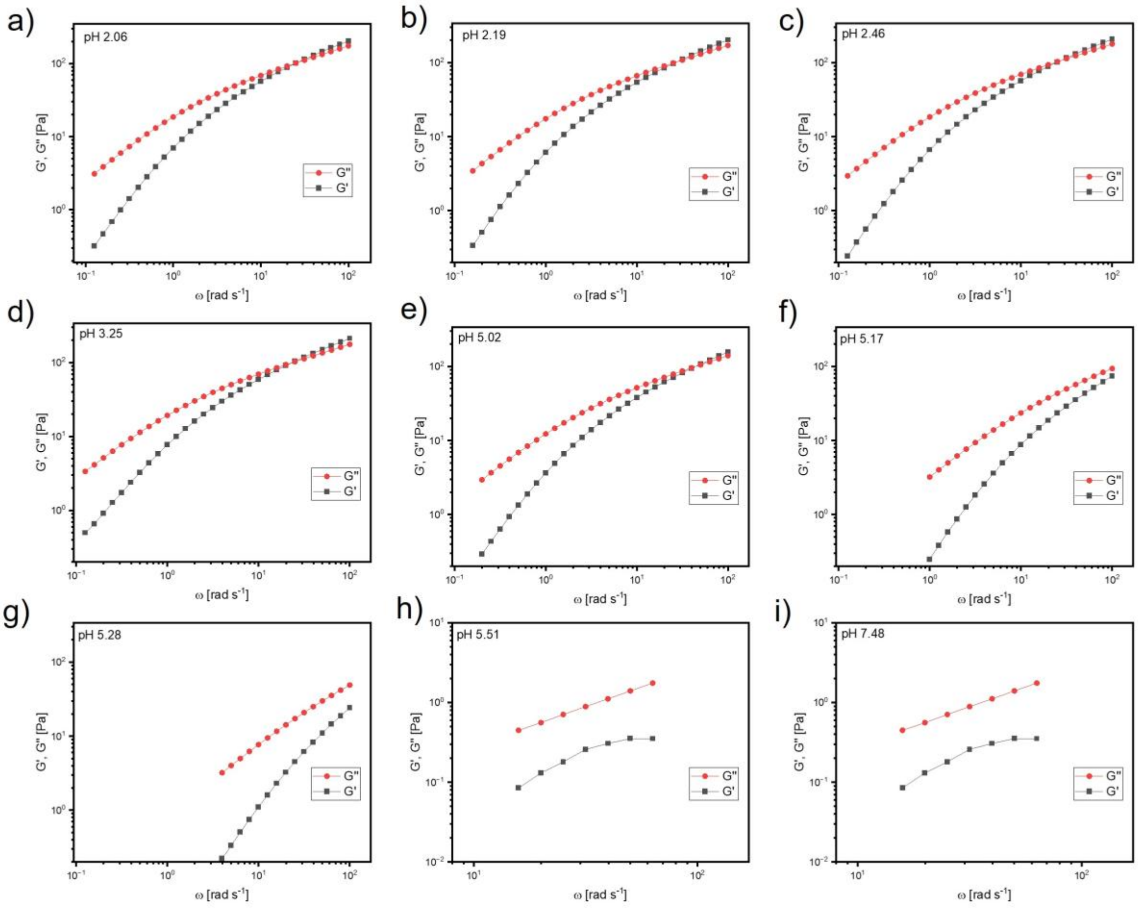

Figure S29. Frequency sweeps of 5 mM PW_{12} in presence of 130 mM HPC in H_2O at pH a) 2.06, b) 2.19, c) 2.46, d) 3.25, e) 5.02, f) 5.17, g) 5.28, h) 5.51 and i) 7.48.

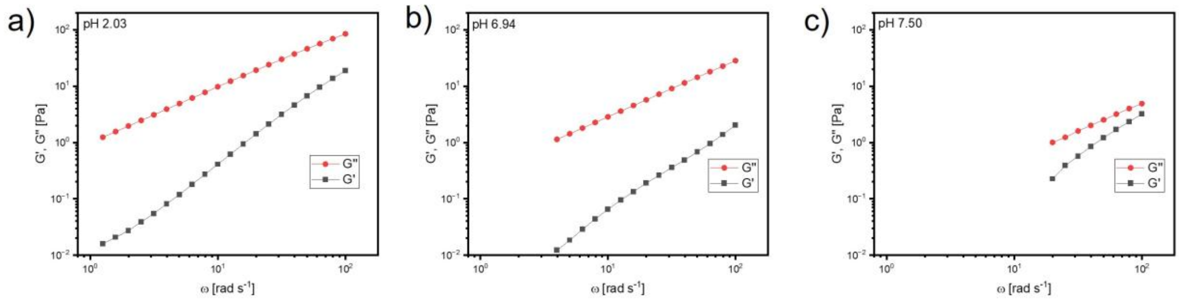

Figure S30. Frequency sweeps of 5 mM SiW_{12} in presence of 130 mM HPC in H_2O at pH a) 2.03, b) 6.94 and c) 7.50.

The crossover relaxation time ($\tau_{crossover}$) was calculated from the frequency sweep by the following equation:

$$\tau_{crossover} = \frac{1}{\omega_{crossover}} \tag{Eq. S33}$$

Where $\omega_{crossover}$ [s^{-1}] is the G'-G'' crossover frequency

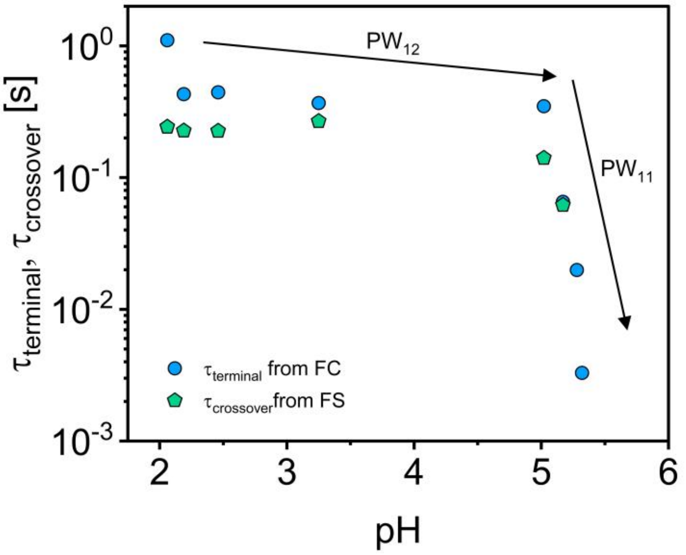

Figure S31. Relaxation time of the 5 mM PW_{12} with 130 mM HPC as determined by flow curves (FC) and frequency sweep (FS).

S10. pH Responsive Solution and Hydrogel

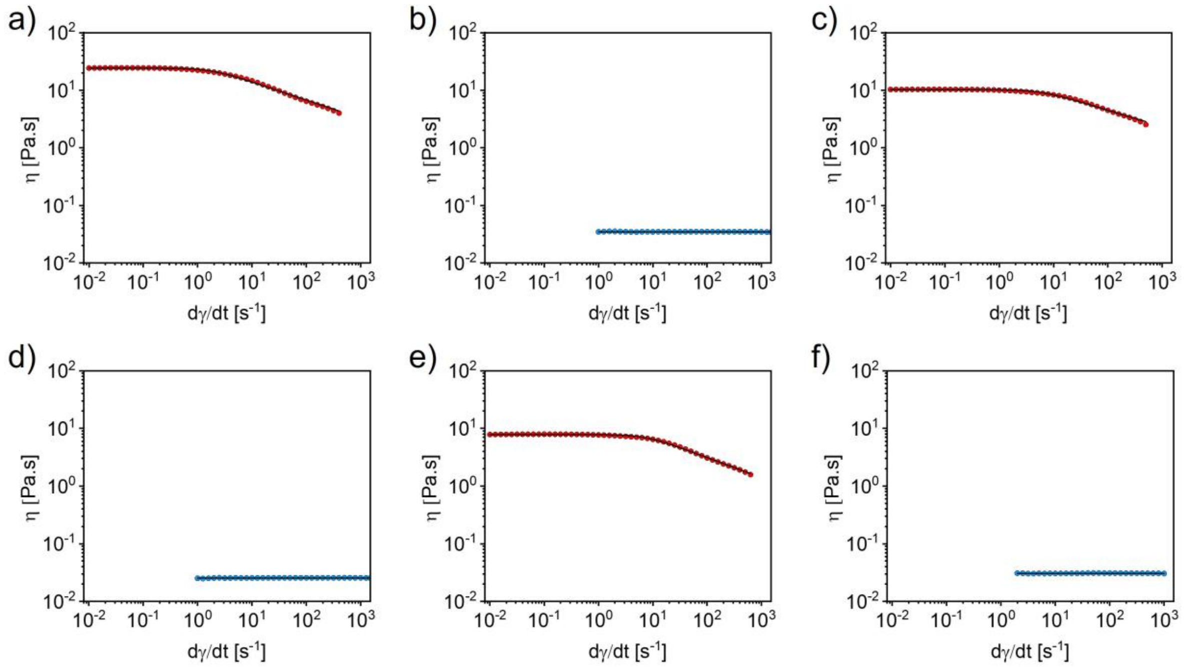

Figure S32. Flow curves of 5 mM PW_{12} with 130 mM HPC showing viscosity changes upon pH switching between pH 2 (red circles symbols, a, c, e) and pH 8 (blue circles, b, d, f). pH adjusted by adding 5 M NaOH / 5 M HCl solution Sample diluted by 1-2% after each step. The black line represents fits using Carreau-Yasuda model.

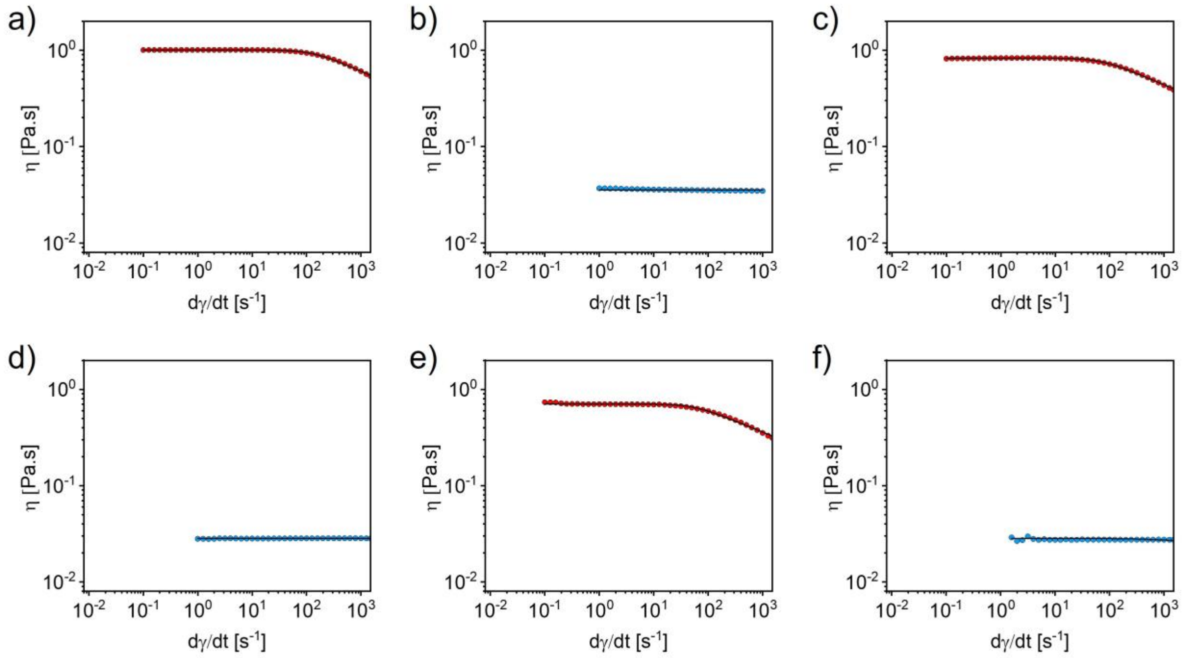

Figure S33. Flow curves of 5 mM SiW_{12} with 130 mM HPC showing viscosity changes upon pH switching between pH 2 (red circles symbols, a, c, e) and pH 9 (blue circles, b, d, f). pH adjusted by adding 5 M NaOH / 5 M HCl solution. Sample diluted by 1-2% after each step. The black line represents fits using Carreau-Yasuda model.

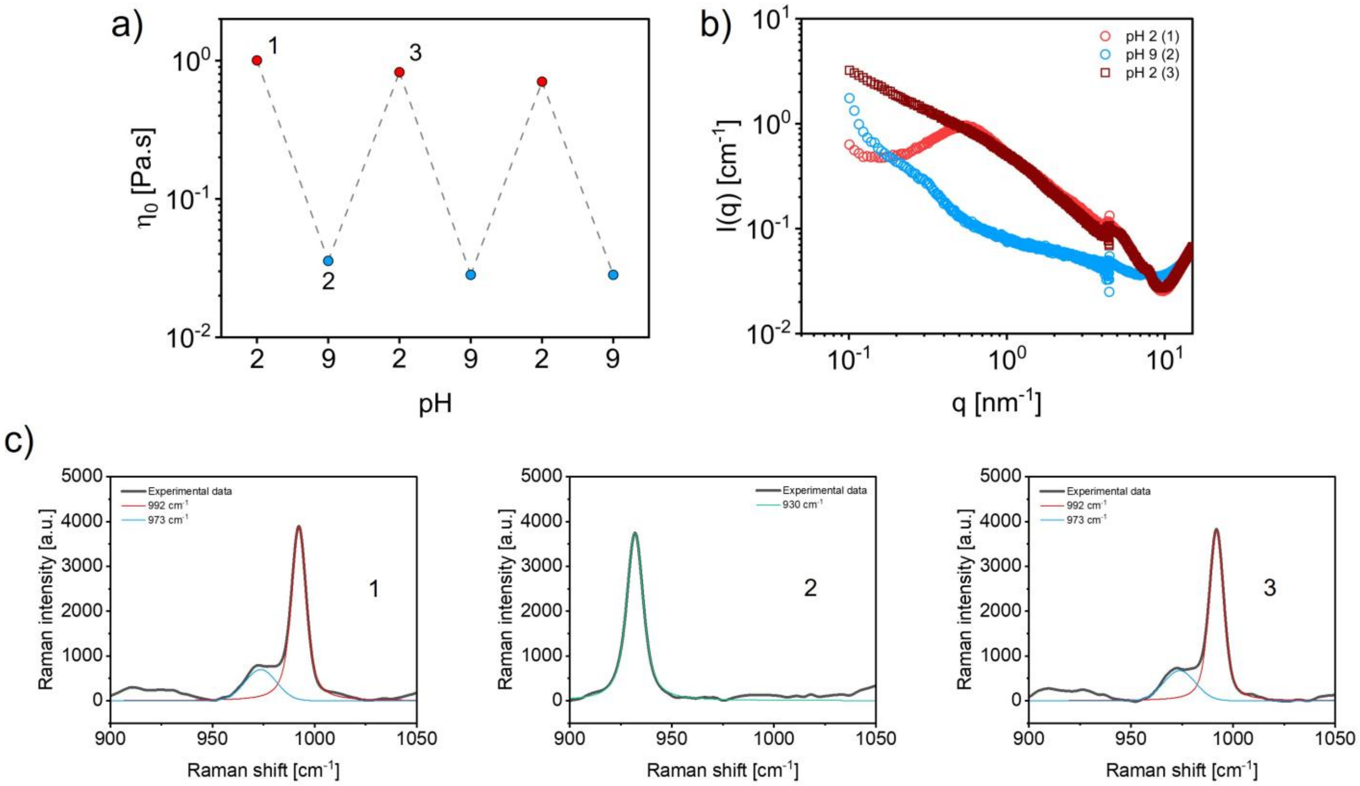

***Figure S34**.* a) pH switching SiW_{12}/HPC solution: a) Zero-shear viscosity (η_0) from flow curves of 130 mM HPC with 5 mM SiW_{12} as a function of pH. b) SAXS profiles and c) Raman spectra of 130 mM HPC with 5 mM SiW_{12} at pH steps 1 to 3. Raman peaks were fitted using Voigt profiles. Peaks at 992 cm^{-1} and 973 cm^{-1} correspond to the plenary SiW_{12}, while the peak at 930 cm^{-1} indicates the presence of tungstate species (WO_4^{2-}).

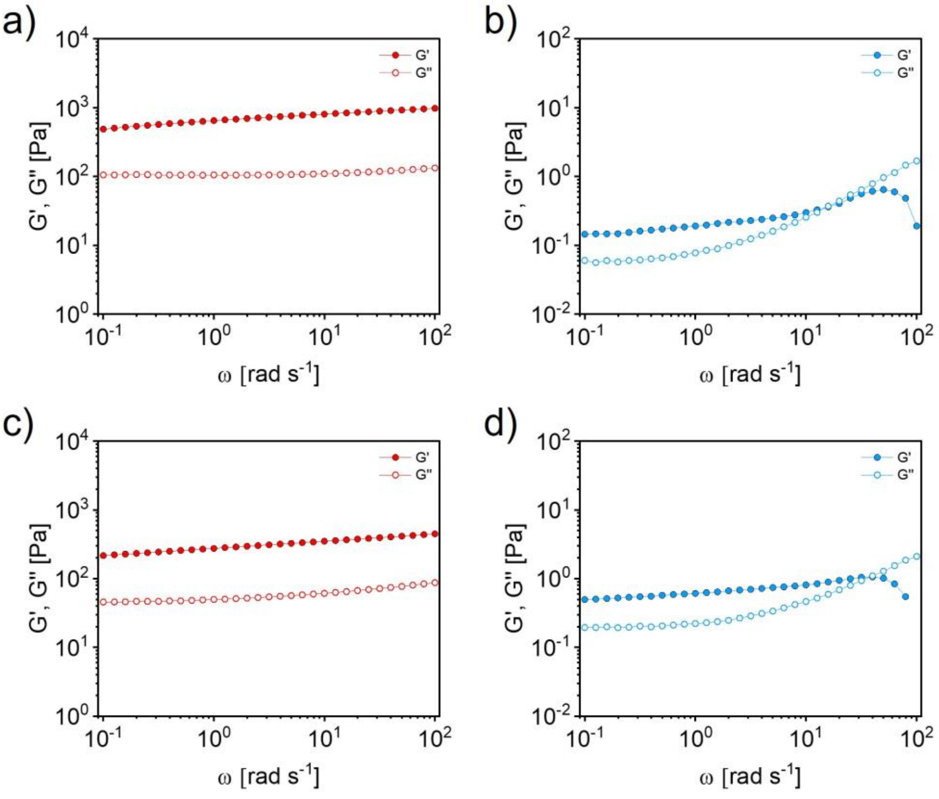

Figure S35. Frequency sweep of 2.5 mM PW_{12} with 130 mM MC showing moduli changes upon pH switching between pH 2 (red circles symbols, a, c) and pH 8 (blue circles, b, d). pH adjusted by adding 5 M NaOH / 5 M HCl solution. Sample diluted by 1-2% after each step. Frequency sweeps measured at 1 % strain within LVER.

S11. References

[1] C. G. Lopez, L. Voleske, W. Richtering, “Scaling laws of entangled polysaccharides” *Carbohydr. Polym.* **2020**, *234*, 115886.

[2] P. Nasatto, F. Pignon, J. Silveira, M. Duarte, M. Noseda, M. Rinaudo, “Methylcellulose, a Cellulose Derivative with Original Physical Properties and Extended Applications” *Polymers* **2015**, *7*, 777–803.

[3] E. Matijević, M. Kerker, “Influence of Electrolytes on the Light Scattering of Inorganic Compounds. Light Scattering of Phosphotungstic Acids[1]” *J. Am. Chem. Soc.* **1959**, *81*, 1307–1310.”

[4] M. Wojdyr, “*Fityk* : a general-purpose peak fitting program” *J. Appl. Crystallogr.* **2010**, *43*, 1126–1128.

[5] L. Alderighi, P. Gans, A. Ienco, D. Peters, A. Sabatini, A. Vacca, “Hyperquad simulation and speciation (HySS): a utility program for the investigation of equilibria involving soluble and partially soluble species” *Coord. Chem. Rev.* **1999**, *184*, 311–318.

[6] C. Falaise, M. A. Moussawi, S. Floquet, P. A. Abramov, M. N. Sokolov, M. Haouas, E. Cadot, “Probing Dynamic Library of Metal-Oxo Building Blocks with γ-Cyclodextrin” *J. Am. Chem. Soc.* **2018**, *140*, 11198–111201.

[7] S. Yao, C. Falaise, N. Leclerc, C. Roch-Marchal, M. Haouas, E. Cadot, “Improvement of the Hydrolytic Stability of the Keggin Molybdo- and Tungsto-Phosphate Anions by Cyclodextrins” *Inorg. Chem.* **2022**, *61*, 4193–4203.

[8] S. Kumar, M. Hoshino, B. Kerkeni, G. García, P. Limão-Vieira, “Isotope Effect in D_2O Negative Ion Formation in Electron Transfer Experiments: DO–D Bond Dissociation Energy” *J. Phys. Chem. Lett.* **2023**, 14, 5362–5369.

[9] R. I. Maksimovskaya, G. M. Maksimov, “^{31}P NMR studies of hydrolytic conversions of 12-tungstophosphoric heteropolyacid” *Coord. Chem. Rev.* **2019**, *385*, 81–99.

[10] R. I. Maksimovskaya, “Hydrolysis of Heteropoly Acid $H_3PW_{12}O_{40}$ by ^{31}P NMR” *Russ. J. Inorg. Chem*. **1998**, *43*, 1825.

[11] P. Dullinger, D. Horinek, “Solvation of Nanoions in Aqueous Solutions” *J. Am. Chem. Soc.* **2023**, *145*, 24922–24930.

[12] X. López, J. A. Fernández, J. M. Poblet, “Redox properties of polyoxometalates: new insights on the anion charge effect” *Dalton Trans.* **2006**, 1162–1167.

[13] D. Franke, M. V. Petoukhov, P. V. Konarev, A. Panjkovich, A. Tuukkanen, H. D. T. Mertens, A. G. Kikhney, N. R. Hajizadeh, J. M. Franklin, C. M. Jeffries, D. I. Svergun, “*ATSAS 2.8* : a comprehensive data analysis suite for small-angle scattering from macromolecular solutions” *J. Appl. Crystallogr.* **2017**, *50*, 1212–1225.

[14] M. R. Antonio, M. K. Bera, “pH-Dependent Interactions between Keggin Heteropolyanions in Dilute Solutions” *Eur. J. Inorg. Chem.* **2019**, *2019*, 367–373.
